# Identification of two recessive etiolation genes (*py1*, *py2*) in pakchoi (*Brassica rapa* L. ssp. *chinensis*)

**DOI:** 10.1186/s12870-020-2271-3

**Published:** 2020-02-10

**Authors:** Kun Zhang, Yu Mu, Weijia Li, Xiaofei Shan, Nan Wang, Hui Feng

**Affiliations:** 1 College of Horticulture, Shenyang Agricultural University, Shenyang, 110866 People’s Republic of China; 2 College of Life Sciences, Shanxi Datong University, Datong, 037009 People’s Republic of China; 3 Institute of Carbon Materials Science, Shanxi Datong University, Datong, 037009 People’s Republic of China

**Keywords:** *Brassica rapa*,, BSR-Seq,, Etiolation mutant,, Genetic mapping

## Abstract

**Background:**

Leaf color is a major agronomic trait, which has a strong influence on crop yields. Isolating leaf color mutants can represent valuable materials for research in chlorophyll (Chl) biosynthesis and metabolism regulation.

**Results:**

In this study, we identified a stably inherited yellow leaf mutant derived from ‘Huaguan’ pakchoi variety via isolated microspore culture and designated as *pylm*. This mutant displayed yellow leaves after germination. Its etiolated phenotype was nonlethal and stable during the whole growth period. Its growth was weak and its hypocotyls were markedly elongated. Genetic analysis revealed that two recessive nuclear genes, named *py1* and *py2*, are responsible for the etiolation phenotype. Bulked segregant RNA sequencing (BSR-Seq) showed that *py1* and *py2* were mapped on chromosomes A09 and A07, respectively. The genes were single Mendelian factors in F_3:4_ populations based on a 3:1 phenotypic segregation ratio. The *py1* was localized to a 258.3-kb interval on a 34-gene genome. The differentially expressed gene *BraA09004189* was detected in the *py1* mapping region and regulated heme catabolism. One single-nucleotide polymorphism (SNP) of *BraA09004189* occurred in *pylm.* A candidate gene-specific SNP marker in 1520 F_3:4_ yellow-colored individuals co-segregated with *py1*. For *py2*, 1860 recessive homozygous F_3:4_ individuals were investigated and localized *py2* to a 4.4-kb interval. Of the five genes in this region, *BraA07001774* was predicted as a candidate for *py2.* It encoded an *embryo defective 1187* and a phosphotransferase related to chlorophyll deficiency and hypocotyl elongation. One SNP of *BraA07001774* occurred in *pylm.* It caused a single amino acid mutation from Asp to Asn. According to quantitative real-time polymerase chain reaction (qRT-PCR), *BraA07001774* was downregulated in *pylm.*

**Conclusions:**

Our study identified a Chl deficiency mutant *pylm* in pakchoi. Two recessive nuclear genes named *py1* and *py2* had a significant effect on etiolation. Candidate genes regulating etiolation were identified as *BraA09004189* and *BraA07001774*, respectively. These findings will elucidate chlorophyll metabolism and the molecular mechanisms of the gene interactions controlling pakchoi etiolation.

## qRT-PCR quantitative real-time PCR

RNA-Seq RNA sequencing

RPKM Reads per kilobases per million mapped reads

SNP Single-nucleotide polymorphism

## Background

The photosynthetic pigment chlorophyll (Chl) is ubiquitous in cyanobacteria and the chloroplasts of higher plants. Chl converts the energy of sunlight into bioavailable chemical energy which drives carbohydrate biosynthesis [1]. Chl is an essential component of leaf color which influences dry matter accumulation and crop yield. In general, leaves appear green because Chl predominates and has a vital role in them. When the Chl content changes in plants, various leaf color mutant phenotypes result including chlorina, virescent, albino, yellow-green, and stay-green [2]. Leaf color mutants develop from the inhibition of genes regulating Chl biosynthesis and chloroplast development. Downregulation of these genes directly or indirectly influences Chl synthesis and degradation and produces the leaf color mutations [3–5]. Thus, leaf color mutants may be ideally suited for the elucidation of the mechanisms of photosynthesis, Chl biosynthesis, chloroplast development, and the expression and regulation of the genes associated with these processes [6–9]. Leaf color mutants have been characterized in *Arabidopsis* [10], rice [11, 12], wheat [13], *Brassica napus* [14], *Brassica oleracea* [15], barley [16], kale [17], tobacco [18], soybean [19], cotton [20], and cucumber [5, 21]. Much research has been invested in the analysis of the genetics, physiology, and molecular mechanisms of Chl biosynthesis and chloroplast development via leaf color mutants.

Several studies in genetic analysis have categorized leaf color mutation inheritance as nuclear and cytoplasmic. Most leaf color mutations are recessively inherited and conferred by a single nuclear gene [21–24]. Leaf color mutations involving two recessive genes are rare. Moreover, their inheritance is complex and it is difficult to apply genetic analysis and gene mapping on them. Wu et al. identified the light color mutant *ws1* in *Nicotiana tabacum* and determined that this phenotype was controlled by the recessive nuclear genes *ws1a* and *ws1b* localized by different BC_1_F_2_ groups to linkage groups 5 and 24, respectively [18]. *BnChd1–1* and *BnChd1–2* are responsible for the light green leaf mutant phenotype in *Brassica napus*. Fine mapping of *BnChd1–1* was achieved using the BC_3_F_1_ population. Candidate gene prediction suggested that *BnChd1–1* encodes a subunit of the nicotinamide adenine dinucleotide phosphate (NADPH) complex in the thylakoid lumen [25]. Chl-deficient mutant phenotypes in durum wheat [26] and *Brassica juncea* [27] are also controlled by two recessive genes. Cytoplasmic mutants are uncommon compared to nuclear mutants. However, they have been reported for tobacco [28], barley [29], and *Brassica campestris* [30].

In plants, Chl biosynthesis comprises 15 enzymatic steps regulated by at least 27 genes [31]. Inactivation mutations of the Chl biosynthetic genes usually results in Chl-deficient mutants [11, 22, 32, 33]. Mutations in the genes governing Chl degradation metabolism generally produce stay-green mutants which retain their green leaf phenotype even during senescence [24, 34, 35]. Chl and heme biosynthesis are two types of tetrapyrrole formation and share a common metabolic pathway from 5-aminolevulinic acid (ALA) to protoporphyrin IX (Proto IX) [36]. Heme is essential for both respiration and photosynthesis. In contrast, excessive heme accumulation inhibits glutamyl-tRNA reductase activity and ALA synthesis, reduces the rate of tetrapyrrole biosynthesis, and affects Chl biosynthesis [37]. Leaf color mutants arising from abnormal heme metabolism were identified for *Arabidopsis* [38], rice [39–41], pea [42], and maize [43].

In a previous study, we developed a pakchoi (*Brassica rapa* L. ssp. *chinensis*) yellow leaf mutant (*pylm*) derived from the ‘Huaguan’ pakchoi variety by isolated microspore culture. This strain is a double haploid (DH) with a stable yellow leaf phenotype [44]. In the present study, we conducted genetic analysis on *pylm* using bulked segregant RNA sequencing (BSR-Seq) with linkage analysis to map the corresponding genes. Then, the candidate genes associated with the mutant phenotype were predicted. The information derived from this work may help facilitate the cloning of etiolation genes and elucidate the molecular mechanisms of gene interactions.

## Results

### Phenotypic characterization of mutant *pylm*

**Fig. 1:**
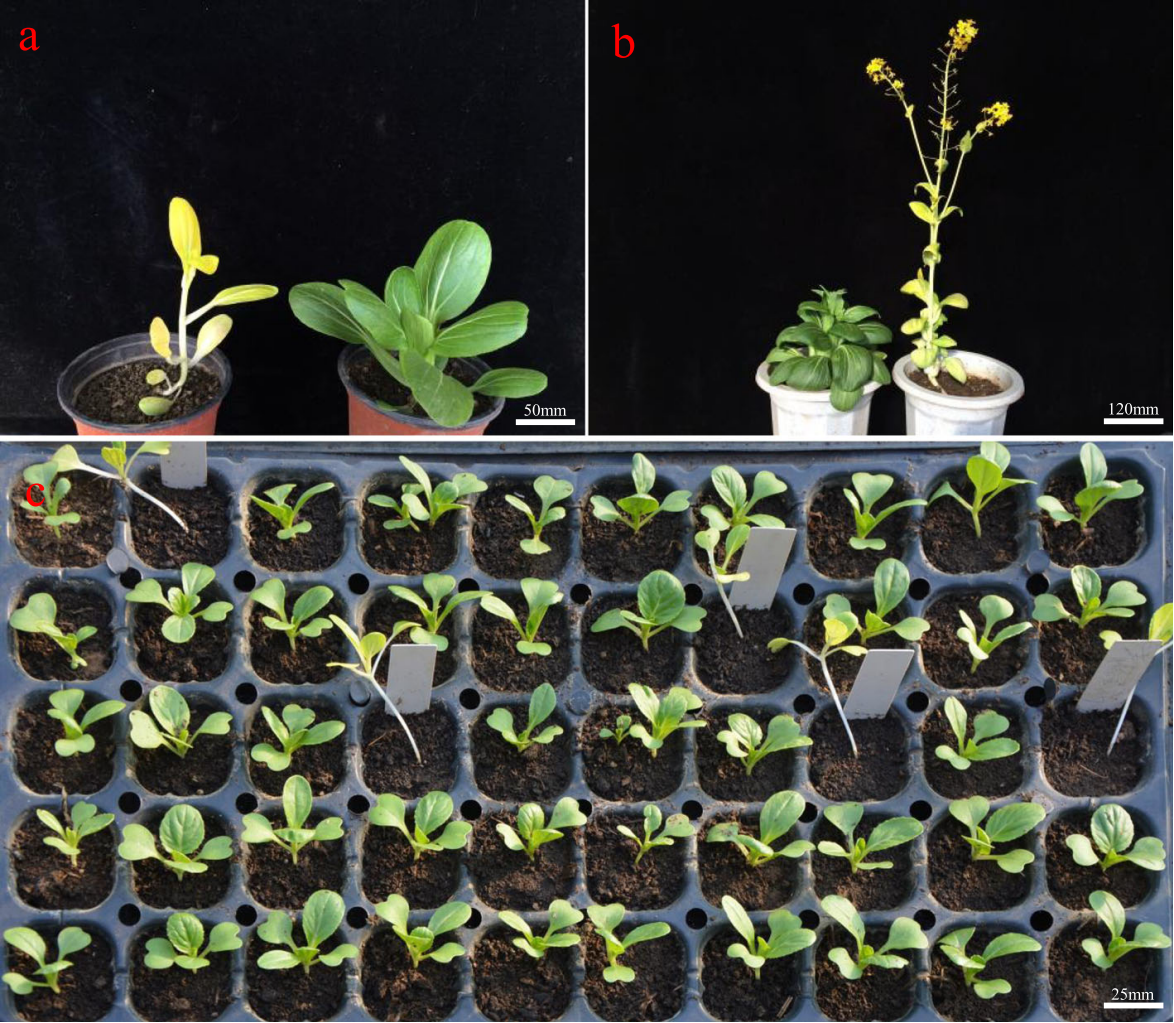
Phenotypes of mutant *pylm* and wild type ‘CK-51’. **a** *pylm* (left) and ‘CK-51’ (right) at the seedling stage; **b** ‘CK-51’ (left) and *pylm* (right) at the bolting stage; **c** Seedling morphology of 2-wk *pylm* and ‘CK-51’ plants. Labeled plants in the tray are *pylm*. Scale bar: a-50 mm; b-120 mm; c-25 mm

The wild type ‘CK-51’ and *pylm* were both obtained from isolated microspore culture of the ‘Huaguan’ pakchoi variety. However, the latter displayed yellow leaves at germination and this phenotype was stable throughout its lifetime (Fig. 1). The mutant had a slender phenotype and weak growth. However, its yellow leaf color was nonlethal. Moreover, relative to ‘CK-51’, *pylm* displayed an elongated hypocotyl at the seedling stage (Fig. 1c) and early flowering at the bolting stage (Fig. 1b).

### Genetic analysis of mutant *pylm*

F_1_ and F_2_ populations were constructed from crosses between *pylm* and the Chinese cabbage DH line ‘FT’ (Additional file 1: Figure S1). The F_1_ individuals from the reciprocal crosses had the same green leaf phenotype as ‘FT’. Therefore, inheritance of the etiolation phenotype in *pylm* is nuclear rather than cytoplasmic. Segregation statistics data for the green and yellow leaf phenotypes of the F_2_ population accorded with the expected Mendelian ratio of 15:1 (χ^2^ < χ^2^_0.05_ = 3.84). Thus, the Chl deficiency trait is controlled by two recessive nuclear genes. The BC_1_ progeny was obtained from F_1_ separately backcrossed with *pylm* and ‘FT’. Segregation statistics data for the green and yellow leaf phenotypes of the BC_1_F_1_ population from the cross of F_1_ with *pylm* fitted the expected Mendelian ratio of 3:1 (χ^2^ < χ^2^_0.05_ = 3.84). This finding confirms that the mutant trait is conferred by two recessive nuclear genes. They were designated as *py1* and *py2*. Neither gene alone can induce the yellow leaf phenotype. Phenotypic data for all generations are listed in Table 1.

**Table 1:** Genetic analysis of leaf color mutant phenotype

Generation	Total	Green-colored	Yellow-colored	Segregation ratio	χ^2^
P_1_ (‘FT’)	92	92	0		
P_2_ (*pylm*)	120	0	120		
F_1_ (P_1_ × P_2_)	258	258	0		
F_1_ (P_2_ × P_1_)	226	226	0		
BC_1_ (F_1_ × ‘FT’)	669	669	0		
BC_1_ (F_1_ × *pylm*)	720	551	169	3.26:1	0.82
F_2_	2376	2243	133	16.86: 1	1.62

### Segregation of *py1* and *py2*

**Fig. 2:**
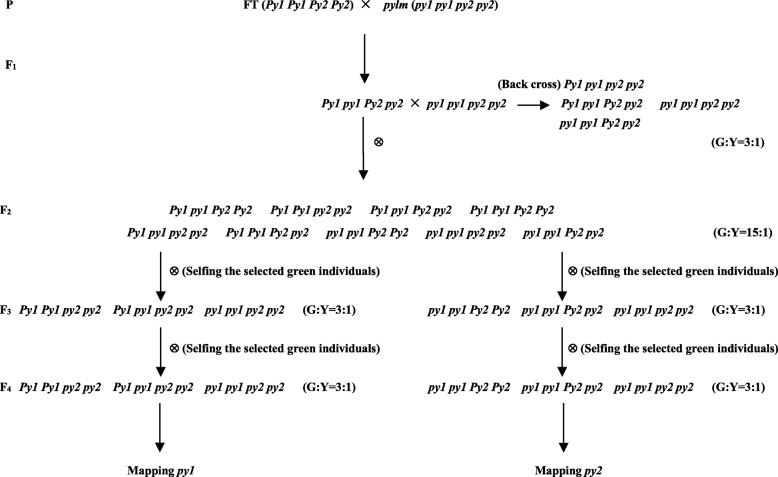
Genetic model for the *pylm* mutant. G and Y indicate green-colored and yellow-colored plants, respectively

According to the genetic analysis of *pylm*, the reduced-Chl phenotype is controlled by the recessive nuclear genes *py1* and *py2*. In consequence, the F_2_ and BC_1_F_1_ populations could not be used to map these genes separately. To separate *py1* from *py2*, F_2_ individuals with green leaves may be randomly selected and self-pollinated to produce F_2:3_ progeny. Green-colored F_2:3_ plants with a statistical segregation ratio of 3:1 (green-colored:yellow-colored) may be self-pollinated to generate F_3:4_ progeny. In theory, ~ 2/3 of the F_3:4_ families should display the expected Mendelian segregation ratio of 3:1. Some of these could map *py1* while the others could map *py2* (Fig. 2).

Twenty green-colored individuals from F_2_ were randomly selected and self-pollinated to produce F_2:3_. For the twenty F_2:3_ families, phenotypic segregations were investigated. There were three distinct groups. Eleven populations showed no yellow-colored plants, five segregated with 15:1, and the other four with 3:1 (Additional file 3: Table S1). These results corroborated the theoretical segregation ratio of “all green-colored:(green-colored:yellow-colored = 3:1):(green-colored:yellow-colored = 15:1) = 7:4:4” for F_2:3_.

Of the four F_2:3_ families segregated with 3:1, eight plants with green leaves per family were selected and self-pollinated to produce F_3:4_. Phenotypic segregations revealed that twenty F_3:4_ families (Nos. 1–20) segregated with 3:1 while the other twelve showed no yellow-colored individuals (Nos. 21–32). Thus, their F_2:3_ genotypes should be *Py1 py1 py2 py2*/*py1 py1 Py2 py2* and *Py1 Py1 py2 py2*/*py1 py1 Py2 Py2*, respectively (Additional file 3: Table S2). Phenotypic segregations of the F_3:4_ families fitted the theoretical segregation ratio of “(green-colored:yellow-colored = 3:1):all green-colored = 2:1”. Therefore, the F_3:4_ families (Nos. 1–20) could be used to map the *py1* and/or *py2* loci.

### BSR-Seq analysis

A total of 47,526,126 and 49,119,466 raw reads (150-bp) were generated from the G-pool and Y-pool, respectively. After quality evaluation and data filtering, 97% of the read pairs (46,456,174 for the G-pool and 47,581,728 for the Y-pool) remained. Clean reads were mapped against the *Brassica* reference genome with Hisat v. 2.0.14. Of these, > 66% were uniquely mapped in both pools.

**Fig. 3:**
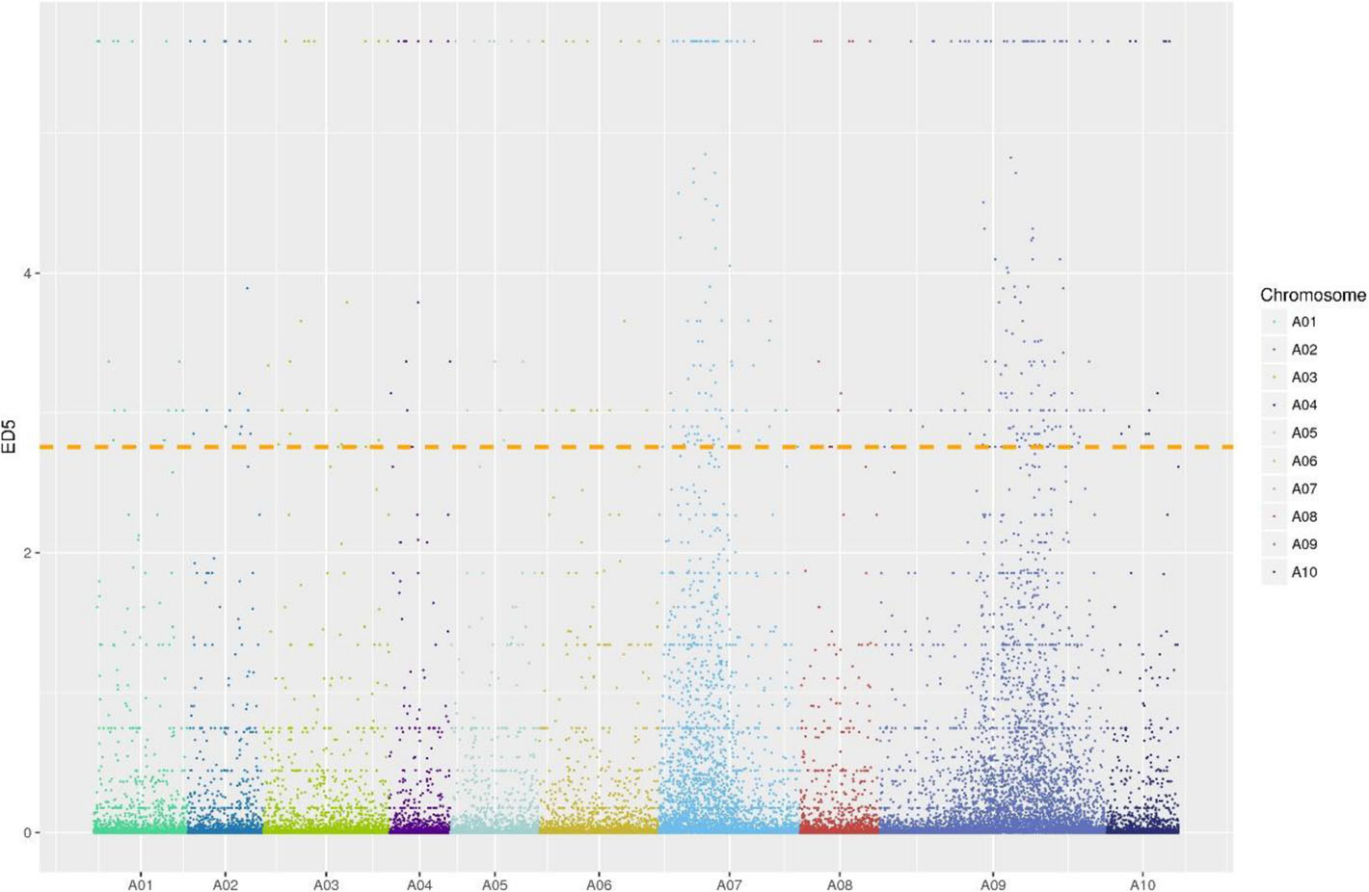
ED^5^ distributions on chromosomes. Each color on the X-axis represents different chromosomes of *Brassica rapa*. Y-axis represents ED^5^ for each differential SNP locus. Horizontal line is the threshold of the top 1% ED^5^

Relative to the reference genome, 154,863 and 157,022 SNPs were detected in the G-pool and Y-pool, respectively. Differential SNP loci were screened for ED^5^ calculation and 412 target differential SNP loci were obtained between the pools according to the top 1% ED^5^ threshold. Two distinct peaks were observed on chromosomes A07 and A09 (Fig. 3). This finding was consistent with the hypothesis that the mutant trait is controlled by two recessive nuclear genes. Thus, it was predicted that the etiolation genes were located on chromosomes A07 and A09 within five chromosome regions (Table 2).

**Table 2:** Localization of chromosome regions related to etiolation genes

Chromosome	Start position	End position	Number of differential SNP loci	Interval length
A07	9,207,067	10,833,976	19	1,626,909
A07	11,475,098	15,522,445	54	4,047,347
A09	19,610,472	20,763,415	11	1,152,943
A09	23,811,435	27,563,122	33	3,751,687
A09	32,067,464	35,505,463	32	3,437,999

### Identification of differentially expressed genes

RPKM was used to measure gene expression level. By setting RPKM ≥0.1, 55,250 genes were detected. These were divided into six RPKM distribution intervals (Additional file 3: Table S3). There were 181 DEGs between the G-pool and Y-pool according to the constraint (|log_2_ fold change| ≥ 1 and FDR ≤ 0.05). Ninety genes were upregulated and the others were downregulated when the G-pool was compared with the Y-pool (Additional file 2: Figure S2). The DEGs are shown in Additional file 3: Table S4.

### Fine mapping of *py1*

Ninety-six SSR markers were developed around the three predicted chromosome regions on chromosome A09. They were used to detect polymorphisms between *pylm* and ‘FT’. After screening, thirty-seven SSR markers displayed polymorphisms between parents. They were used to test twelve green-colored and yellow-colored individuals each from the No. 1 F_3:4_ family. SSRzk5 and SSRzk12 were located near the 23,811,435-27,563,122 region on chromosome A09 and showed linkage to *py1* on the opposite side.

**Fig. 4:**
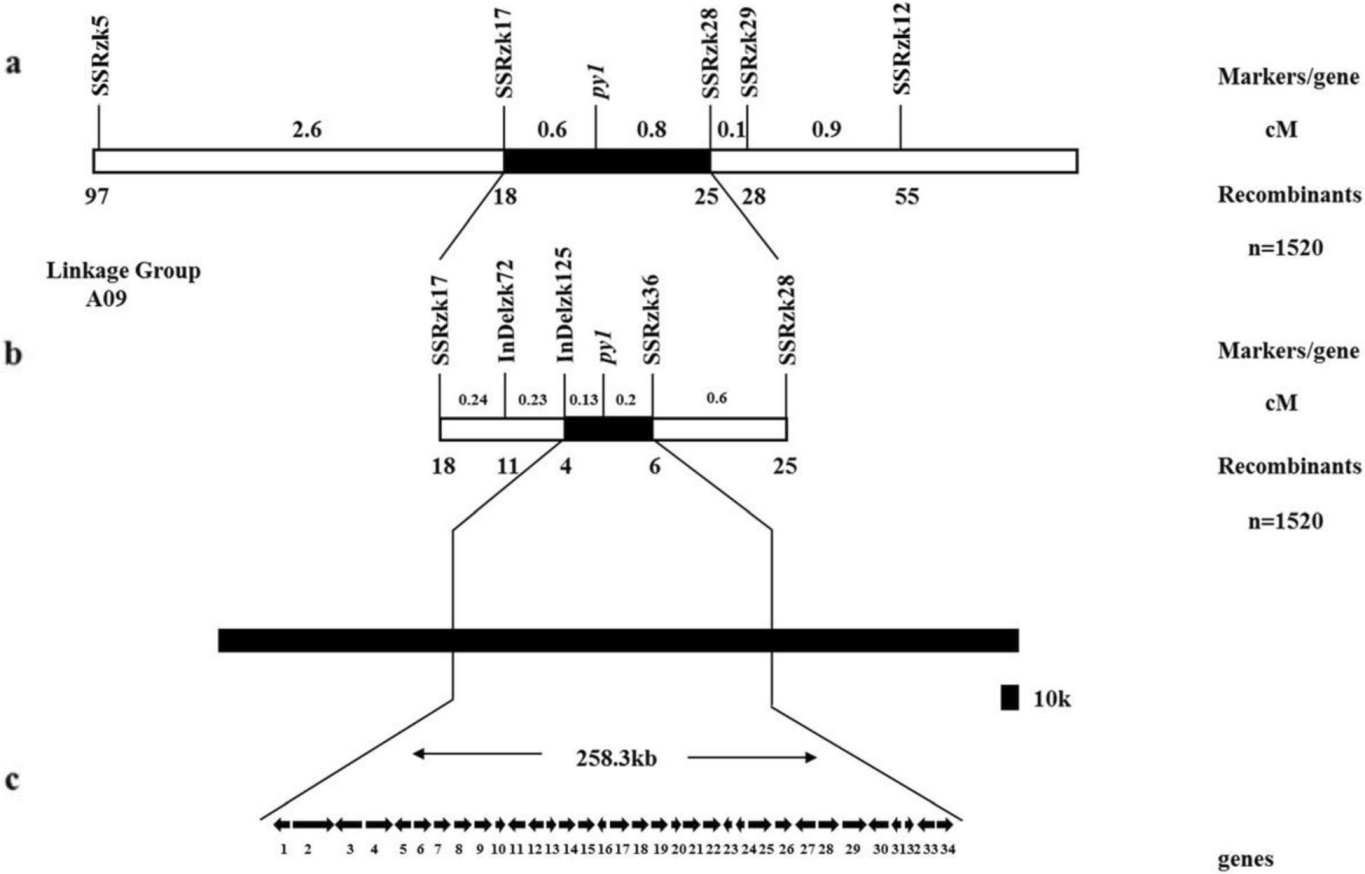
Genetic and physical *py1* maps and candidate gene analysis. **a**: Chromosome A09 linkage map was constructed with 1520 *pylm* individuals from the No. 1 F_3:4_ family. The *py1* was preliminarily mapped between SSRzk17 and SSRzk28; **b**: Fine mapping of *py1*. The *py1* was restricted to the region between Indelzk125 and SSRzk36. Number of recombinants between the markers and *py1* is shown below the genetic map. The mapping distance above the linkage map is in centimorgan (cM) units; **c**: Candidate *py1* region and the annotated genes in the *Brassica* database. The *py1* locus was narrowed to a 258.3-kb region comprising 34 predicted genes. The numbers 1–34 refer to the candidate genes. The arrows indicate the direction of gene expression. Detailed information on the 34 genes is presented in Additional file 3: Table S6

A total of 1520 yellow-colored individuals of the No. 1 F_3:4_ family were selected as the *py1* mapping population. A linkage analysis disclosed that *py1* was located between SSRzk5 and SSRzk12 at estimated genetic distances of 3.2 cM and 1.8 cM, respectively (Fig. 4a). To identify the molecular markers tightly linked to *py1* and narrow the *py1* mapping interval, new SSR and Indel markers were developed between SSRzk5 and SSRzk12. The polymorphic markers SSRzk17, SSRzk28, SSRzk29, SSRzk36, Indelzk72, and Indelzk125 were linked to *py1* (Additional file 3: Table S5). SSRzk17, Indelzk72, and Indelzk125 were located on one side of *py1* as SSRzk5 while SSRzk28, SSRzk29, and SSRzk36 were located on the other side of *py1* as SSRzk12. The *py1* was mapped between Indelzk125 and SSRzk36 at 0.13 cM and 0.2 cM, respectively (Fig. 4b). Therefore, *py1* was mapped in a 258.3-kb region between the most tightly linked markers (Fig. 4c).

### Candidate *py1* analysis

The target DNA sequences of the 258.3-kb region between Indelzk125 and SSRzk36 were obtained from the *Brassica* database. A genomic sequence analysis revealed that the candidate region contained 34 genes (Fig. 4c, Additional file 3: Table S6). Differential gene expression analysis disclosed only *BraA09004189* in the *py1* mapping region. *BraA09004189* is a heme oxygenase (HO1) which participates in heme catabolism. Mutants with yellow leaf phenotype induced by defective HOs were reported in earlier studies [40, 41]. *BraA09004189* was predicted to be the most probable candidate *py1* gene.

**Fig. 5:**
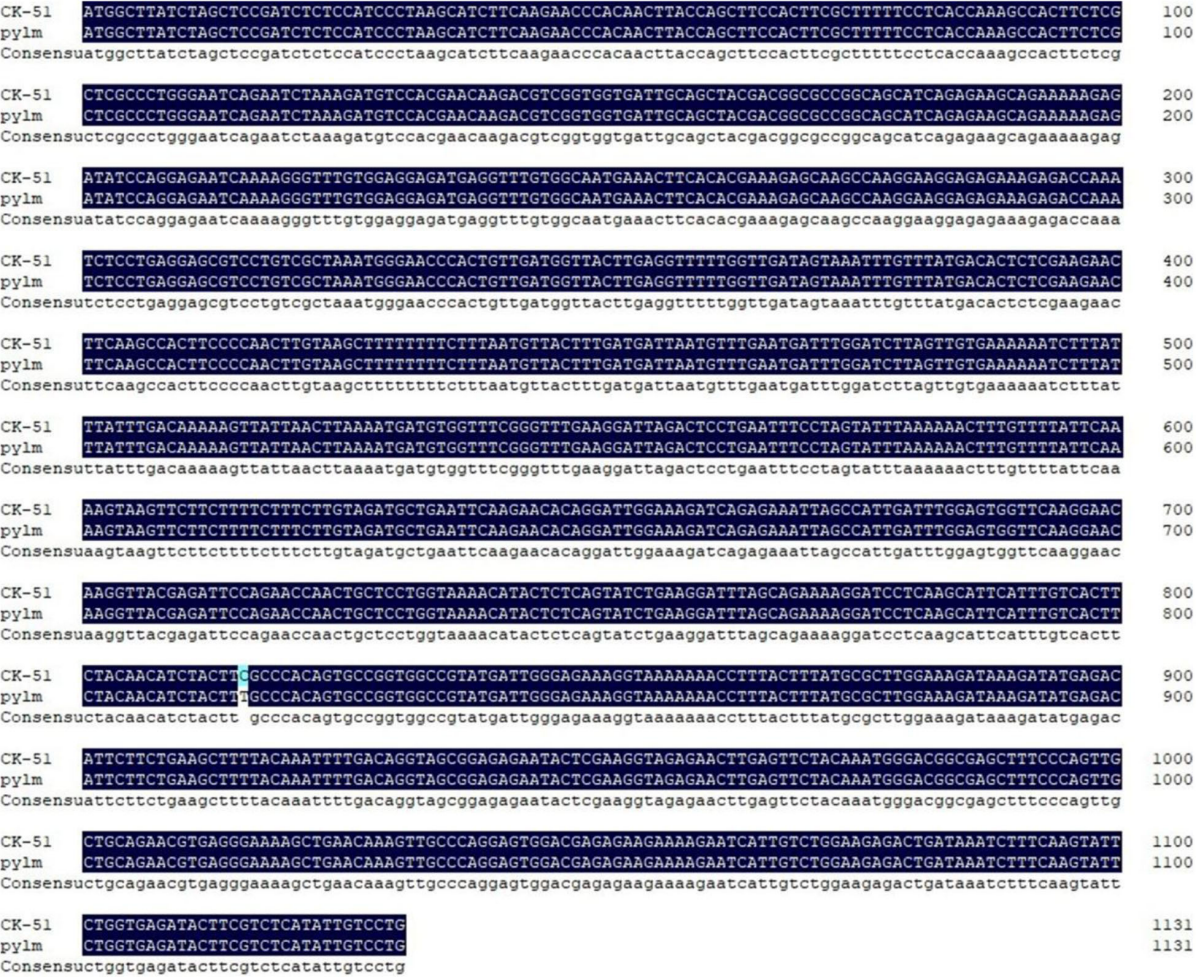
Sequence alignments of *BraA09004189* in ‘CK-51’ and *pylm*

To confirm this hypothesis, two pairs of primers were designed to sequence *BraA09004189* in *pylm* and ‘CK-51’ (Additional file 3: Table S7). The BraA sequence did not differ between parents whereas the BraB sequence in *pylm* presented with one SNP (Fig. 5). Based on the position of *BraA09004189*, an SNP marker was designed to screen 1520 yellow-colored individuals from the No. 1 F_3:4_ family. The bands of whole mapping individuals co-segregated with *py1*.

**Fig. 6:**
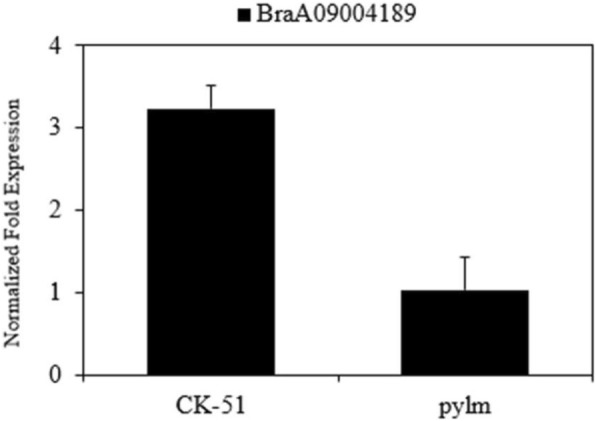
*BraA09004189* expression analyses by qRT-PCR for *pylm* and ‘CK-51’. Error bars indicate standard errors of the means of three replicates

A qRT-PCR was performed to determine *BraA09004189* expression in *pylm* and ‘CK-51’. In accordance with the differential gene expression analysis, the results indicated that *BraA09004189* expression level was much higher in ‘CK-51’ than that in *pylm* (Fig. 6). This finding further supports the likelihood that *BraA09004189* is the candidate for *py1*.

### Fine mapping of *py2*

Considering the constructed populations size, we screened the Nos. 2–5 F_3:4_ families using the same research strategy applied for SSRzk5 and SSRzk12 linked to *py1*. The etiolation gene *py1* was identified in the Nos. 2, 4, and 5 F_3:4_ families. In theory, then, the No. 3 F_3:4_ family may be used to establish the *py2* locus.

**Fig. 7:**
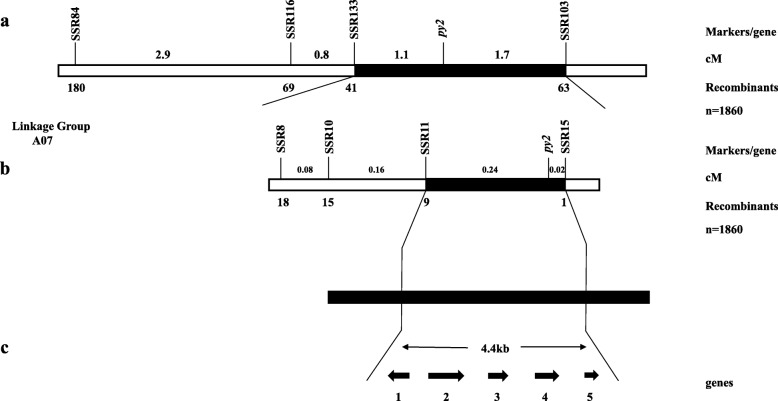
Genetic and physical maps of *py2* and candidate gene analysis. **a**: Linkage map of chromosome A07 was constructed with 1860 individuals bearing the *pylm* phenotype in the No. 3 F_3:4_ family. The *py2* was preliminarily mapped between SSR133 and SSR103; **b**: Fine mapping of *py2*. The *py2* was restricted to the region between SSR11 and SSR15. The number of recombinants between the markers and *py2* is shown under the genetic map. The distance above the linkage map is in centimorgan (cM) units; **c**: Candidate *py2* region and annotated genes in the *Brassica* database. The *py2* locus was narrowed to a 4.4-kb region containing the five predicted genes *BraA07001773*-*BraA07001777*. Arrows indicate the direction of gene expression. Detailed information on these genes is presented in Table S9

Forty-eight SSR markers were developed around the two predicted regions on chromosome A07 to detect polymorphisms between *pylm* and ‘FT’. After screening, eleven SSR markers displayed polymorphisms between the parents. They were used to test twelve green-colored and twelve yellow-colored individuals of the No. 3 F_3:4_ family. SSR84 and SSR103 were located around the region 11,166,810-15,034,483 on chromosome A07 and presented with linkages to *py2* on the opposite side (Fig. 7a; Additional file 3: Table S8).

There were 1860 yellow-colored individuals from the No. 3 F_3:4_ family selected as the *py2* mapping population. The *py2* was located between SSR11 and SSR15 at estimated genetic distances of 0.24 cM and 0.02 cM, respectively (Fig. 7b). To narrow the *py2* mapping interval and identify the molecular markers tightly linked to *py2*, new SNP markers were developed between SSR11 and SSR15. Only the polymorphic marker SNP11 was linked to *py2*. Based on the recombinant individuals, the *py2* interval was narrowed to 14,851,951–14,896,902 and contained five genes (Fig. 7c).

### Candidate *py2* analysis

**Fig. 8:**
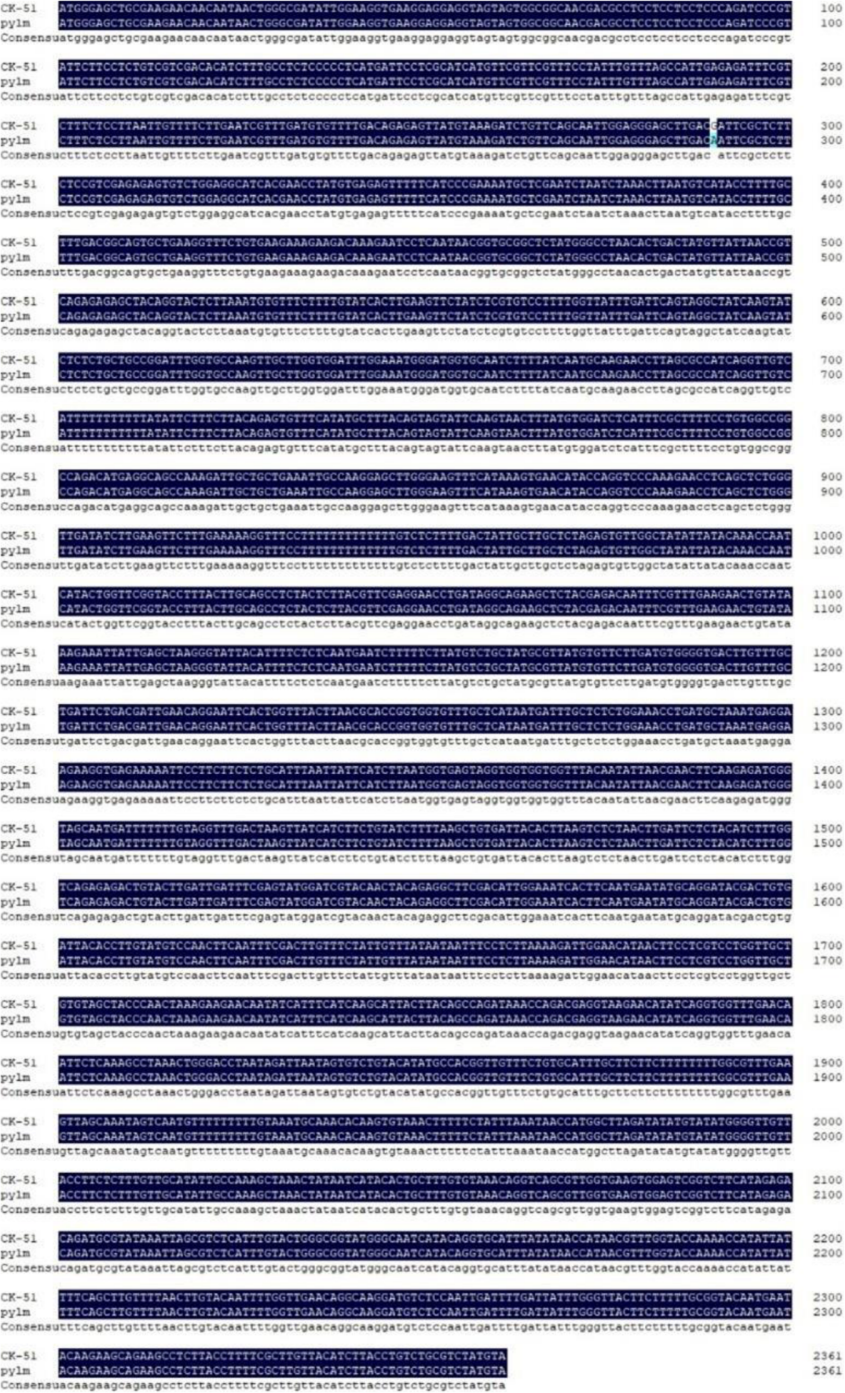
Sequence alignments of *BraA07001774* in ‘CK-51’ and *pylm*

**Fig. 9:**
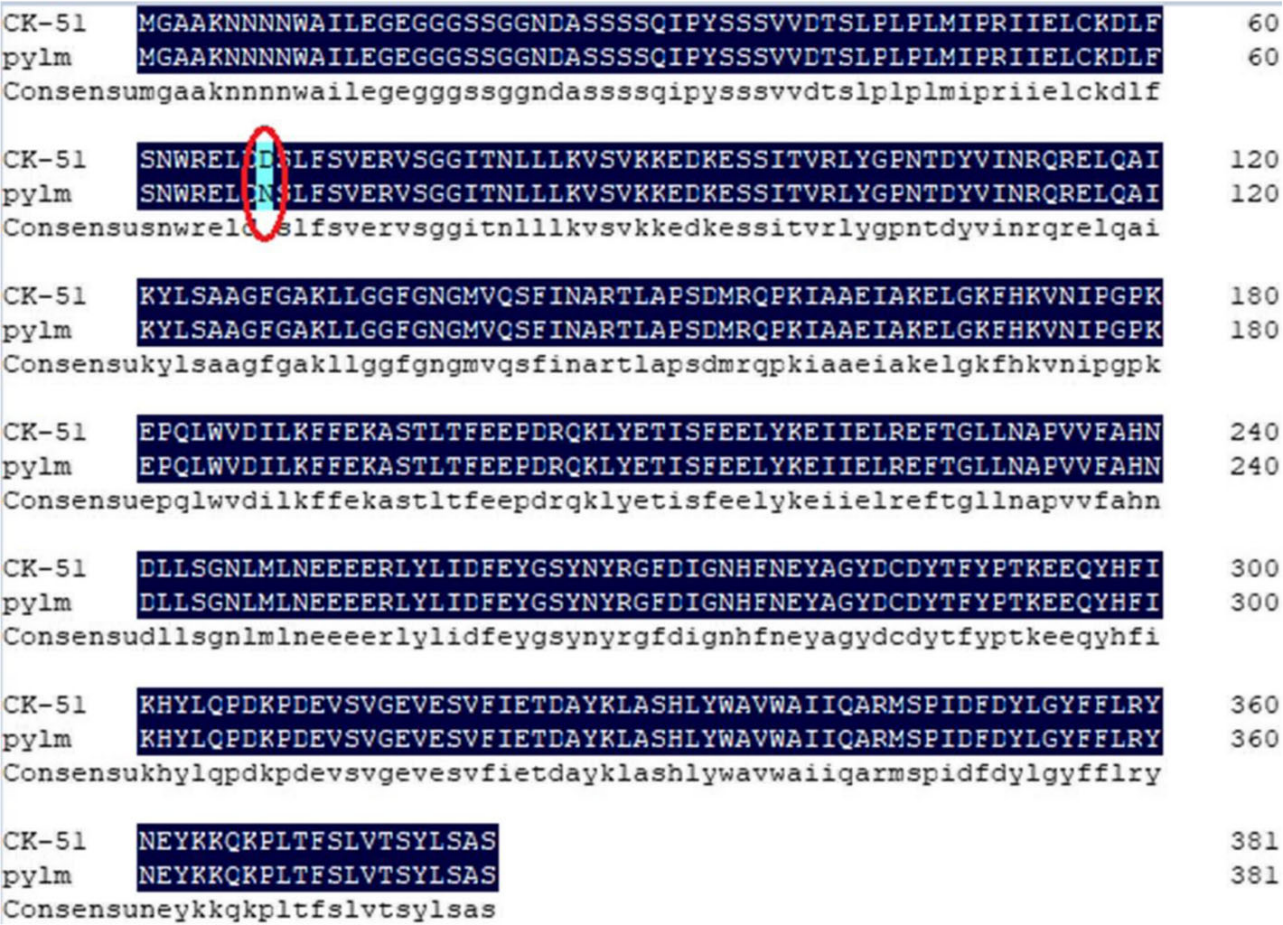
Amino acid sequence alignments of *BraA07001774* in ‘CK-51’ and *pylm*

Annotation data for the five candidate genes in the *py2* target region were obtained from the *Brassica* database (Additional file 3: Table S9). Primers were designed to cover the cDNA for each gene and predict the candidate genes (Additional file 3: Table S10). There were no differences between *pylm* and ‘CK-51’ in terms of *BraA07001775*, *BraA07001776*, or *BraA07001777*. After PCR amplification, the *BraA07001773* sequence was disordered and the sequence comparisons were inconsistent over serial repetitions. There was SNP variation between parents for the first exon in *BraA07001774* (Fig. 8). It caused a single amino acid mutation from Asp (GAT) in the wild type to Asn (AAT) in *pylm* (Fig. 9). Therefore, *BraA07001774* was taken as the most probable candidate gene for *py2*.

**Fig. 10:**
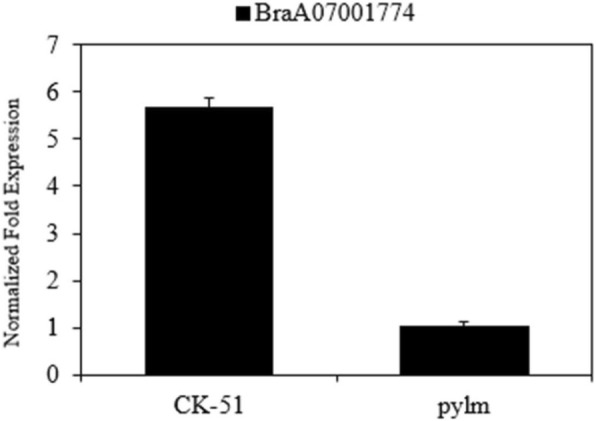
Expression analysis of *BraA07001774* in *pylm* and ‘CK-51’ by qRT-PCR. Error bars indicate standard errors of the means of three replicates

*BraA07001774* is an *embryo defective 1187* (*emb 1187*) and a phosphotransferase. The albino mutants (*pds1*, *pds2*) phenotypes in *Arabidopsis thaliana* may be caused by *emb 71* [45]. For *Arabidopsis* seeds with silique defects, hypocotyl elongation was characterized during the development of F_2_ generation mutant seedlings [46]. We proposed that the mutant phenotype is determined by mutations in *BraA07001774*. To validate our prediction, *BraA07001774* expression in *pylm* and ‘CK-51’ was analyzed by qRT-PCR. *BraA07001774* was dramatically downregulated in *pylm* (Fig. 10). Thus, it probably is the candidate gene for *py2*.

## Discussion

Mutations in leaf color are widespread in nature. The main type of leaf color mutation is Chl deficiency. Dwarfism, retarded growth, attenuated photosynthetic capacity, low yield, and death are associated with this defect [25, 47, 48]. Here, we identified the pakchoi yellow leaf mutant *pylm* from isolated microspore culture. Unlike previously reported Chl deficient mutants, *pylm* presented with substantially elongated hypocotyls at the seedling stage and early flowering at the bolting stage. The etiolation phenotype in *pylm* was nonlethal and stable throughout the growth period. The Chl deficiency in *pylm* was controlled by two recessive genes. These characteristics suggested that *pylm* was of high value for research in revealing the Chl biosynthesis mechanism regulated by gene interactions.

Map-based cloning is an effective gene isolation strategy. It has been extensively used for plant gene function analysis [49–51]. However, it is contingent upon fine mapping of the target gene. For most leaf color mutants, the traits are recessively inherited and controlled by a single nuclear gene. The F_2_ populations are instinctively applied to map the target gene [5, 41, 48]. With regard to the character conferred by two recessive nuclear genes, F_2_ populations may also be used in preliminary mapping. An efficient way to isolate allele pairs from each other and separately map them is to construct advanced backcrosses and other populations. The recessive white stem (*ws*) loci in *Nicotiana tabacum* and the Chl deficiency (*Bnchd1*) loci in *Brassica napus* were successfully mapped using constructed BC_1_F_2_ and BC_3_F_1_ populations, respectively [18, 25]*.* In the present study, genetic analysis revealed that the recessive nuclear genes designated as *py1* and *py2* were responsible for the etiolation trait. We successfully segregated *py1* from *py2* and constructed an inheritance model for the Chl deficiency trait in pakchoi. Twenty F_3:4_ families with a phenotypic segregation ratio of 3:1 were constructed. Various F_3:4_ families were successfully used to map the *py1* and *py2* loci separately. Compared to using advanced backcross populations to map pairs of recessive nuclear genes, creating and using F_2:3_ or F_3:4_ families avoid the selection errors and interference in genetic analysis caused by the incomplete emasculation of *Brassica rapa*.

BSR-Seq efficiently combines the respective superiorities of bulk segregation analysis (BSA) and RNA sequencing (RNA-Seq) for rapid gene mapping [52–55]. BSR-Seq is targeted at the mRNA level. It selects phenotypically opposite individuals from segregated populations and constructs two RNA mixing pools to find SNPs at the transcript level. The transcriptome data localize the target genes and detect potentially associated DEGs [56]. BSR-Seq has been extensively applied to map the causal genes linked to a single target trait [24, 57, 58]. Two independently inherited traits may also be localized by BSR-Seq. Tan et al. applied BSR-Seq to locate the genes controlling male sterility on chromosome A05 and white petal on chromosome A02 [59]. Here, the mutant *pylm* and Chinese cabbage DH ‘FT’ lines were chosen as parents to construct the F_2_ separation population. The phenotypes of the wild type and mutant individuals significantly differed. Thus, extreme mixed pools could be accurately and conveniently created for BSR-Seq. Release of *Brassica rapa* genomic data enhanced the reliability of these populations in BSR-Seq applications. Five candidate regions related to the yellow leaf phenotype in *pylm* were identified on chromosomes A07 and A09. Molecular markers were developed according to the locations of the candidate regions. The etiolation genes *py1* and *py2* were separately mapped with different F_3:4_ families. The findings confirmed the feasibility of BSR-Seq for mapping two recessive nuclear genes. They also showed that BSR-Seq simplifies molecular marker development and screening in traditional mapping methods and greatly improves their efficiency.

New molecular markers were developed near the target regions based on preliminary *py1* mapping by BSR-Seq. The *py1* was mapped between the markers Indelzk125 and SSRzk36 on A09 chromosome over a 258.3-kb localization interval containing 34 predicted genes. No new polymorphic SSR or Indel markers were available to limit the localization interval. The gene expression patterns determined by BSR-Seq disclosed only one differentially expressed gene (*BraA09004189*) within the *py1* mapping region. A gene annotation referenced from the *Brassica* database indicated that *BraA09004189* encodes heme oxygenase-1 (HO1). This enzyme plays a vital role in phytochrome chromophore metabolism, the photoresponse mechanism, adventitious root formation, and oxidative damage mitigation [60–63]. HO1 stabilizes and maintains the heme content by transforming heme into BV-IXα [64]. As embranchments of tetrapyrrole biosynthesis, Chl and heme biosynthesis share a common metabolic pathway from ALA to Proto IX. Excessive heme accumulation caused by abnormal heme metabolism leads to feedback inhibition of Chl biosynthesis [36]. Therefore, a decrease in HO1 activity may influence Chl biosynthesis. The *hy1* mutant of *Arabidopsis* and the *yellow-green leaf 2* mutant of rice presented with the reduced-Chl phenotype because of free heme inhibition resulting from HO1 mutations [40, 60]. HO1 defects strongly affected thylakoid development in rice [41]. Davis et al. found that the abnormally elongated hypocotyl phenotype of mutant *Arabidopsis* seedlings may be associated with HO1 defects [60]. Thus, *py1* may encode HO and a mutation thereof may influence Chl biosynthesis and leaf color. In this study, qRT-PCR demonstrated that *BraA09004189* was downregulated in *pylm.* This finding was consistent with those obtained by BSR-Seq. The SNP *BraA09004189* was detected between *pylm* and ‘CK-51’. A candidate gene-specific SNP marker in 1520 F_3:4_ yellow-colored individuals co-segregated with *py1*. Thus, *BraA09004189* corresponds to the yellow leaf locus *py1* in *pylm*.

It was already known that certain Chl deficiency traits are controlled by two recessive nuclear genes. However, there was a lack of appropriate mapping populations or reliable molecule markers. Therefore, they were either approximately mapped without definite locations [18, 26] or only one of the pair could be localized [25]. In previous studies, little progress was made in the simultaneous fine mapping or accurate prediction of the candidate genes. Here, we used the same mapping strategy as that for *py1* to accomplish fine mapping for *py2*. The linkage analysis disclosed that *py2* was mapped between SSR11 and SSR15 on chromosome A07. The mapping interval was narrowed to 4.4 kb by the SNP11 marker linked to *py2.* Sequence analysis of the five genes in the *py2* localization interval showed that only *BraA07001774* expression significantly differed between *pylm* and ‘CK-51’. For *pylm*, *BraA07001774* had a SNP missense mutation on the first exon such that the wild type had an Asp residue whereas *pylm* had an Asn. The qRT-PCR revealed that *BraA07001774* was downregulated in the mutant relative to the wild type*.* Gene annotation in the *Brassica* database indicated that *BraA09004189* encodes *emb 1187* and a phosphotransferase. In *Arabidopsis*, the *emb* genes are essential for seed development [65]. *EMB* genes encode various proteins. Thirty percent of them are active in the plastids [66]. Most *emb* mutations result in albinism or etiolated seeds and embryos which are secondary effects of mutations in chloroplast biogenesis and function [67]. The albino mutants (*pds1*, *pds2*) and hypocotyl elongation phenotypes in *Arabidopsis* may be related to mutations in *EMB* genes [45, 46]. Thus, *BraA07001774* is a candidate gene for *py2.*

## Conclusions

We reported the identification of a Chl deficiency mutant *pylm* in pakchoi in this study. The etiolation trait was controlled by two recessive nuclear genes *py1* and *py2*. We successfully segregated *py1* from *py2* by constructing F_3:4_ families and achieved fine mapping and predictions for the two etiolation genes. Candidate genes regulating etiolation were identified as *BraA09004189* and *BraA07001774*, respectively. These discoveries may help elucidate the molecular mechanisms underlying the trait controlled by two recessive nuclear genes. In future studies, functional validation will be conducted to clarify the functions of these candidate genes. In this manner, the molecular mechanism of gene interactions may be better understood.

## Methods

### Plant materials and mapping population development

The DH line *pylm* was obtained by isolated microspore culture of the ‘Huaguan’ pakchoi variety introduced from Japan musashino seed company. This strain was characterized by yellow leaves and elongated hypocotyls [44]. The parent used for the segregating population development with *pylm* in this study was ‘FT’, a DH line derived from Chinese cabbage variety ‘Fukuda 50’ screened by Shenyang greenstar Chinese cabbage research institute (Shenyang, China), which exhibits folded green leaves [68]. The *pylm* was reciprocally crossed with ‘FT’ to produce the F_1_, F_2_, and BC_1_ generations. Twenty F_2_ individuals with green leaves were self-pollinated to produce F_2:3_ progenies. Eight green F_2:3_ individuals per group were randomly selected from the corresponding populations and self-pollinated to produce the F_3:4_ populations. Those with character segregation were used in linkage analysis and gene mapping. All plants were grown in the greenhouse at Shenyang Agricultural University, China.

### Detection of variations by BSR-Seq

One hundred individuals with extreme leaf color phenotype at three-leaf stage were separately collected from the F_2_ progeny and pooled for RNA extraction. Total RNA of each sample was extracted using TRIzol Reagent (Invitrogen, USA). The RNA concentration and integrity were analyzed with an Agilent 2100 Bioanalyzer (Agilent Technologies, USA). The extreme mixed pools green-leaf (G-pool) and yellow-leaf (Y-pool) were constructed by mixing equal amounts of each RNA sample. RNA-Seq library preparations were constructed according to the manufacturer’s protocol (NEBNext® Ultra™ RNA Library Prep Kit for Illumina®). Sequencing was run on an Illumina HiSeq 2500 by GENEWIZ Suzhou Biological Technology Co., Ltd., China.

The quality of the raw RNA-Seq reads was assessed with FastQC (v. 0.10.1). Adapter sequences and low-quality reads containing N and < 70 were deleted from the raw reads with Cutadapt (v. 1.9.1). Low-quality bases at the 5′ or 3′ end were filtered out. Those with mean quality < 20 were trimmed by the 4-bp sliding window method. Clean data were aligned to the *Brassica* database (http://brassicadb.org/brad/) with Hisat v. 2.0.14 [69]. Candidate single nucleotide polymorphisms (SNPs) between the pools were obtained with the Mpileup module in SAM Tools (v. 0.1.18) SNP loci with depth coverage > 3× were screened for differential SNP analysis in the mutant and wild type pools. Euclidean distances (ED) for the differential SNP loci were calculated. The ED for each differential SNP locus was raised to the power five, namely, ED^5^, to eliminate background noise [70]. All ED^5^ were sorted and the differential SNP loci with ED^5^ in the top 1% were screened and mapped to specific chromosome regions based on the SNP locus distributions. Chromosome regions associated with the target traits were predicted according to the distributions of the ED^5^ for the differential SNP loci on the chromosomes.

### Differential gene expression analysis

To detect differentially expressed genes (DEGs) between the pools, a gene expression level analysis was performed with Htseq (v. 0.6.1). The reads per kilobases per million mapped reads (RPKM) were calculated [71]. DEGs were screened using a preset threshold (|log_2_ fold change| ≥ 1 and false discovery rate (FDR) ≤ 0.05).

### DNA isolation, polymerase chain reaction (PCR), and linkage analysis

A modified cetyltrimethylammonium bromide (CTAB) method [72] was used to extract genomic DNA from young leaves of the parental and F_3:4_ populations with a phenotypic segregation ratio of 3:1. The primers for polymorphism analysis were designed in Primer Premier (v. 5.0). PCR amplifications were performed following the instruction as described by Wang et al. [24]. PCR products were separated on 5% (w/v) denaturing polyacrylamide gels and examined by silver staining. The genetic linkage map was constructed by Join Map v. 4.0 [73] using the segregation data. Map distances were calculated and reported in centimorgans (cM) according to Kosambi’s mapping function [74].

### Candidate gene prediction and quantitative real-time PCR (qRT-PCR) validation

Using the chromosome location of the target gene, all adjacent genes were annotated with the *Brassica* database (http://brassicadb.org/brad/). Candidate genes were predicted according to gene annotation. PCR primers were designed to span the entire putative gene length between the mutant *pylm* and wild type ‘CK-51’. The control line ‘CK-51’, with a green leaf phenotype, was obtained in the same manner as the *pylm* mutant during the same period [44]. Candidate genes were cloned following the method referenced from Huang et al. [75]. Sequences were determined by GENEWIZ Biological Technology Co. Ltd. (Suzhou, China) and aligned with DNAMAN software.

Leaves of *pylm* and ‘CK-51’ were collected for total RNA extraction as previously described. After confirming its concentration and integrity, the RNA was reverse-transcribed with a FastQuant RT Kit (Tiangen, China). qRT-PCR was performed with a Bio-Rad IQ5 Real-Time PCR System (Bio-Rad Laboratories, USA) and SYBR Green PCR Master Mix (Tiangen, China). All reactions were run on three biological replicates. Two independent technical replicates per sample were processed to confirm data accuracy. Primers for the candidate genes *BraA09004189* (F: 5′-GCTTCCACTTCGCTTTTTCCT-3′; R: 5′-TCTTTTTCTGCTTCTCTGATGCTG-3′) and *BraA07001774* (F: 5′-GGATACGACTGTGATTACACCTTCTAC-3′; R: 5′-CCGACTCCACTTCACCAACG-3′) were used in the qRT-PCR analysis. The *Actin* gene (F: 5′-CGAAACAACTTACAACTCCA-3′; R: 5′-CTCTTTGCTCATACGGTCA-3′) served as an endogenous control. The qRT-PCR reaction conditions and program were those cited in Huang et al. [76]. The relative expression level was calculated by the 2^−ΔΔCt^ method [77]. Bio-Rad IQ5 (Bio-Rad Laboratories, USA) analyzed the data.

## Supplementary information

**Additional file 1: Figure S1.** Phenotypes of the *pylm* mutant in pakchoi (left) and the DH line ‘FT’ in Chinese cabbage (right). Scale bar: 40 mm

**Additional file 2: Figure S2.** DEGs between the G-pool and the Y-pool (|log_2_ fold change| ≥ 1 and FDR ≤ 0.05)

**Additional file 3: Table S1.** Phenotypic segregation ratios of the F_2:3_ populations and their F_2_ genotypes from the cross ‘FT’ × *pylm.* **Table S2.** Phenotypic segregation ratios of the F_3:4_ populations and their F_2:3_ genotypes from the cross ‘FT’ × *pylm.* **Table S3.** RPKM interval distribution of the 55,250 genes identified in the G-pool and Y-pool via BSR-Seq. **Table S4.** Gene identification and expression data for the DEGs in the Y-pool vs. the G-pool. **Table S5.** Primer sequences for the SSR and Indel markers tightly linked with *py1*. **Table S6.** Prediction of candidate genes within the gene-mapped region on chromosome A09. **Table S7.** Sequences of the primers used to clone the full-length and CDS sequences of *BraA09004189.* **Table S8.** Primer sequences of the SSR markers tightly linked with *py2.* **Table S9.** Prediction of candidate genes within the gene-mapped region on chromosome A07. **Table S10.** Sequences of the primers used to cloning the full-length sequences of the candidate genes for *py2*

## Data Availability

The datasets analysed during the current study are available in the *Brassica* database (http://brassicadb.org/brad/). All data used and/or analyzed are available upon request.

## Abbreviations

‘FT’: Fukuda 50
ALA: 5-Aminolevulinic acid
BSA: Bulk segregation analysis
BSR-Seq: Bulked segregant RNA sequencing
Chl: Chlorophyll
cM: Centimorgans
CTAB: Cetyltrimethylammonium bromide
DEGs: Differentially expressed genes
DH: Double haploid
ED: Euclidean distances
*emb*: *embryo defective*
FDR: False discovery rate
HO1: Heme oxygenase-1
NADPH: Nicotinamide adenine dinucleotide phosphate
PCR: Polymerase chain reaction
Proto IX: Protoporphyrin IX
*pylm*: Pakchoi (*Brassica rapa* L. ssp. *chinensis*) yellow leaf mutant
qRT-PCR: quantitative real-time PCR
RNA-Seq: RNA sequencing
RPKM: Reads per kilobases per million mapped reads
SNP: Single-nucleotide polymorphism
