# Supplementary figures and images for: Identification of two recessive etiolation genes (py1, py2) in pakchoi (Brassica rapa L. ssp. chinensis)
Source: BMC Plant Biol. 2020 Feb 10;20:68. doi: 10.1186/s12870-020-2271-3 (PMC7011377; doi:10.1186/s12870-020-2271-3)

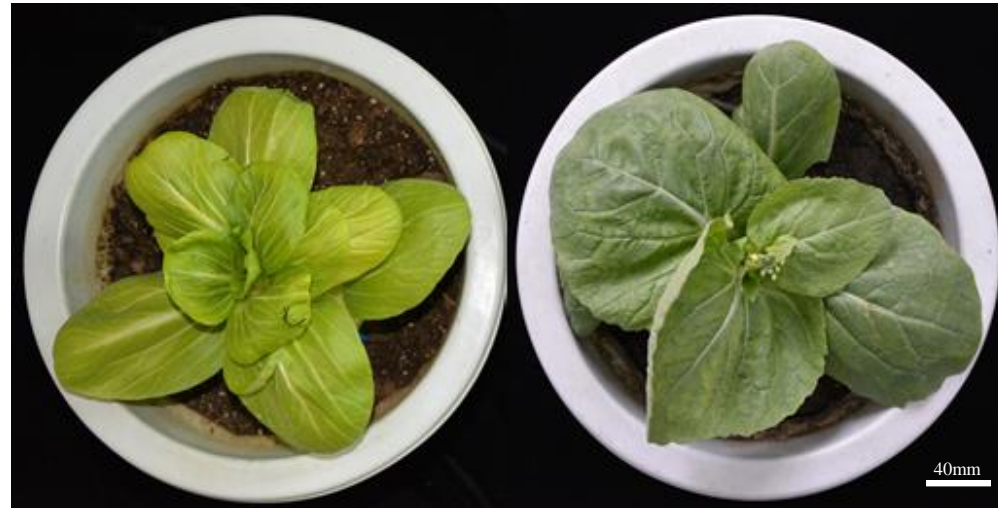

Supplement: Supplementary file 1 — Additional file 1: Figure S1. Phenotypes of the pylm mutant in pakchoi (left) and the DH line ‘FT’ in Chinese cabbage (right). Scale bar: 40 mm [file 12870_2020_2271_MOESM1_ESM.pdf]

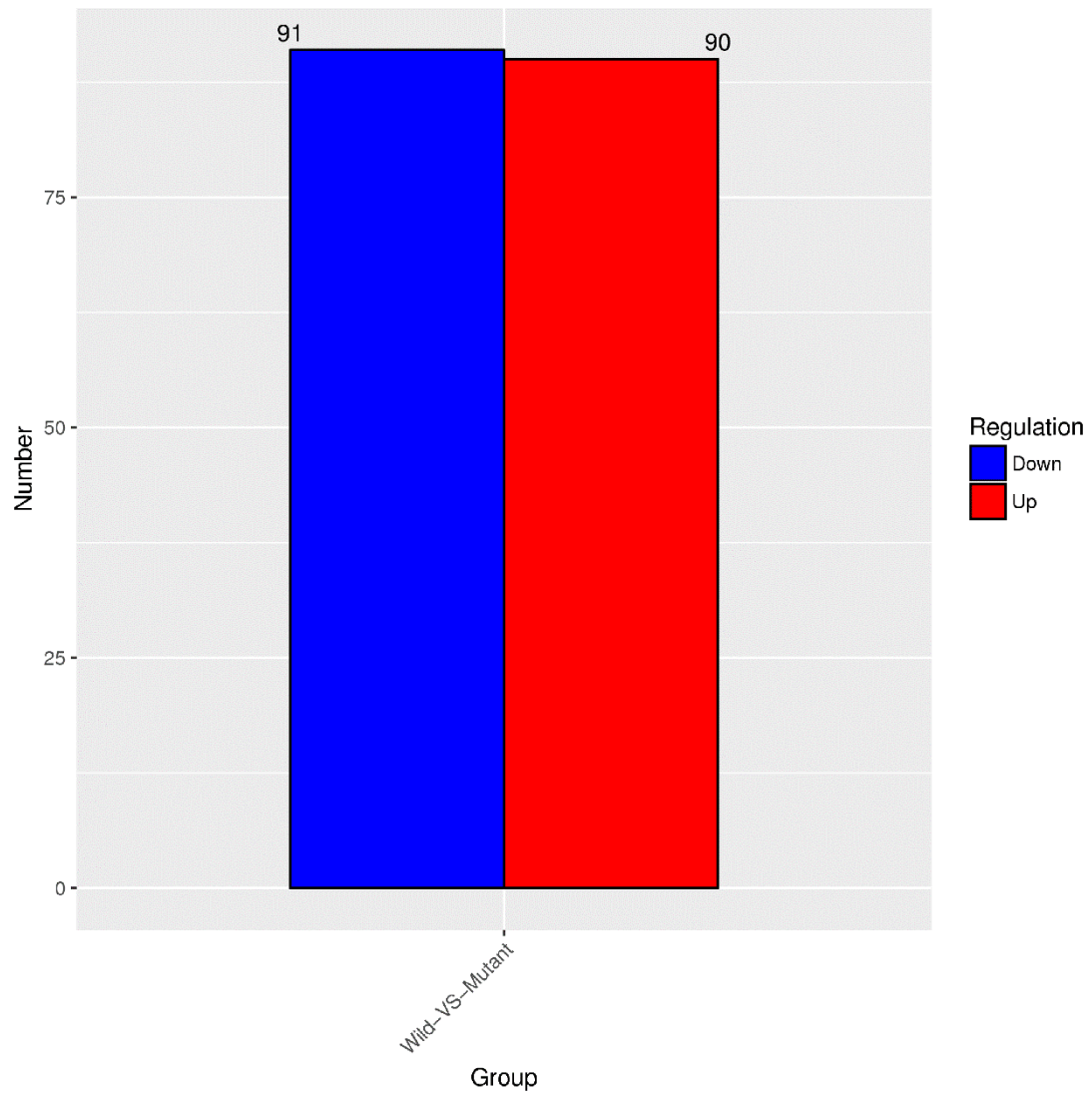

Supplement: Supplementary file 2 — Additional file 2: Figure S2. DEGs between the G-pool and the Y-pool (|log2 fold change| ≥ 1 and FDR ≤ 0.05) [file 12870_2020_2271_MOESM2_ESM.pdf]
