# Supplementary material for: Identification of two recessive etiolation genes (py1, py2) in pakchoi (Brassica rapa L. ssp. chinensis)
Source: BMC Plant Biol. 2020 Feb 10;20:68. doi: 10.1186/s12870-020-2271-3 (PMC7011377; doi:10.1186/s12870-020-2271-3)
Supplement: Supplementary file 3 — Additional file 3: Table S1. Phenotypic segregation ratios of the F2:3 populations and their F2 genotypes from the cross ‘FT’ × pylm. Table S2. Phenotypic segregation ratios of the F3:4 populations and their F2:3 genotypes from the cross ‘FT’ × pylm. Table S3. RPKM interval distribution of the 55,250 genes identified in the G-pool and Y-pool via BSR-Seq. Table S4. Gene identification and expression data for the DEGs in the Y-pool vs. the G-pool. Table S5. Primer sequences for the SSR and Indel markers tightly linked with py1. Table S6. Prediction of candidate genes within the gene-mapped region on chromosome A09. Table S7. Sequences of the primers used to clone the full-length and CDS sequences of BraA09004189. Table S8. Primer sequences of the SSR markers tightly linked with py2. Table S9. Prediction of candidate genes within the gene-mapped region on chromosome A07. Table S10. Sequences of the primers used to cloning the full-length sequences of the candidate genes for py2 [file 12870_2020_2271_MOESM3_ESM.docx]

**Table S1** Phenotypic segregation ratios of the F_2:3_ populations and their F_2_ genotypes from the cross ‘FT’ × *pylm*

| Number | Green-colored plants | Yellow-colored plants | Total | Segregation ratio | χ^2^ test | F_2_ Genotype |
| --- | --- | --- | --- | --- | --- | --- |
| 1 | 100 | 44 | 144 | 2.27: 1 | 2.08 | *Py1py1py2py2*/ *py1py1Py2py2* |
| 2 | 112 | 50 | 162 | 2.24: 1 | 2.67 | *Py1py1py2py2/ py1py1Py2py2* |
| 3 | 108 | 36 | 144 | 3.00: 1 | 0.01 | *Py1py1py2py2/ py1py1Py2py2* |
| 4 | 108 | 42 | 150 | 2.57: 1 | 0.57 | *Py1py1py2py2/ py1py1Py2py2* |
| 5 | 122 | 0 | 122 |  |  | *Py1Py1Py2Py2/Py1Py1Py2py2/ Py1Py1py2py2/py1py1Py2Py2/ Py1py1Py2Py2* |
| 6 | 144 | 0 | 144 |  |  | *Py1Py1Py2Py2/Py1Py1Py2py2/ Py1Py1py2py2/py1py1Py2Py2/ Py1py1Py2Py2* |
| 7 | 140 | 0 | 140 |  |  | *Py1Py1Py2Py2/Py1Py1Py2py2/ Py1Py1py2py2/py1py1Py2Py2/ Py1py1Py2Py2* |
| 8 | 156 | 0 | 156 |  |  | *Py1Py1Py2Py2/Py1Py1Py2py2/ Py1Py1py2py2/py1py1Py2Py2/ Py1py1Py2Py2* |
| 9 | 132 | 0 | 132 |  |  | *Py1Py1Py2Py2/Py1Py1Py2py2/ Py1Py1py2py2/py1py1Py2Py2/ Py1py1Py2Py2* |
| 10 | 138 | 0 | 138 |  |  | *Py1Py1Py2Py2/Py1Py1Py2py2/ Py1Py1py2py2/py1py1Py2Py2/ Py1py1Py2Py2* |
| 11 | 143 | 0 | 143 |  |  | *Py1Py1Py2Py2/Py1Py1Py2py2/ Py1Py1py2py2/py1py1Py2Py2/ Py1py1Py2Py2* |
| 12 | 127 | 0 | 127 |  |  | *Py1Py1Py2Py2/Py1Py1Py2py2/ Py1Py1py2py2/py1py1Py2Py2/ Py1py1Py2Py2* |
| 13 | 158 | 0 | 158 |  |  | *Py1Py1Py2Py2/Py1Py1Py2py2/ Py1Py1py2py2/py1py1Py2Py2/ Py1py1Py2Py2* |
| 14 | 136 | 0 | 136 |  |  | *Py1Py1Py2Py2/Py1Py1Py2py2/ Py1Py1py2py2/py1py1Py2Py2/ Py1py1Py2Py2* |
| 15 | 150 | 0 | 150 |  |  | *Py1Py1Py2Py2/Py1Py1Py2py2/ Py1Py1py2py2/py1py1Py2Py2/ Py1py1Py2Py2* |
| 16 | 162 | 14 | 176 | 11.57: 1 | 0.61 | *Py1py1Py2py2* |
| 17 | 160 | 12 | 172 | 13.33: 1 | 0.06 | *Py1py1Py2py2* |
| 18 | 147 | 12 | 159 | 12.25: 1 | 0.26 | *Py1py1Py2py2* |
| 19 | 154 | 11 | 165 | 14.00: 1 | 0.00 | *Py1py1Py2py2* |
| 20 | 164 | 13 | 177 | 12.62: 1 | 0.20 | *Py1py1Py2py2* |

**Table S2** Phenotypic segregation ratios of the F_3:4_ populations and their F_2:3_ genotypes from the cross ‘FT’ × *pylm*

| Number | Green-colored plants | Yellow-colored plants | Total | Segregation ratio | χ^2^ test | F_3_ Genotype |
| --- | --- | --- | --- | --- | --- | --- |
| 1 | 55 | 21 | 76 | 2.62: 1 | 0.16 | *Py1py1py2py2*/*py1py1Py2py2* |
| 2 | 66 | 20 | 86 | 3.30: 1 | 0.06 | *Py1py1py2py2*/*py1py1Py2py2* |
| 3 | 48 | 15 | 63 | 3.20: 1 | 0.01 | *Py1py1py2py2*/*py1py1Py2py2* |
| 4 | 43 | 12 | 55 | 3.58: 1 | 0.33 | *Py1py1py2py2*/*py1py1Py2py2* |
| 5 | 56 | 11 | 67 | 5.09: 1 | 2.19 | *Py1py1py2py2*/*py1py1Py2py2* |
| 6 | 75 | 17 | 92 | 4.41: 1 | 1.75 | *Py1py1py2py2*/*py1py1Py2py2* |
| 7 | 65 | 16 | 81 | 4.06: 1 | 0.93 | *Py1py1py2py2*/*py1py1Py2py2* |
| 8 | 43 | 11 | 54 | 3.91: 1 | 0.40 | *Py1py1py2py2*/*py1py1Py2py2* |
| 9 | 70 | 17 | 87 | 4.12: 1 | 1.11 | *Py1py1py2py2*/*py1py1Py2py2* |
| 10 | 55 | 14 | 69 | 3.93: 1 | 0.58 | *Py1py1py2py2*/*py1py1Py2py2* |
| 11 | 63 | 18 | 81 | 3.50: 1 | 0.20 | *Py1py1py2py2*/*py1py1Py2py2* |
| 12 | 59 | 17 | 76 | 3.47: 1 | 0.16 | *Py1py1py2py2*/*py1py1Py2py2* |
| 13 | 78 | 19 | 97 | 4.11: 1 | 1.24 | *Py1py1py2py2*/*py1py1Py2py2* |
| 14 | 50 | 14 | 64 | 3.57: 1 | 0.19 | *Py1py1py2py2*/*py1py1Py2py2* |
| 15 | 54 | 18 | 72 | 3.00: 1 | 0.02 | *Py1py1py2py2*/*py1py1Py2py2* |
| 16 | 45 | 14 | 59 | 3.21: 1 | 0.01 | *Py1py1py2py2*/*py1py1Py2py2* |
| 17 | 57 | 16 | 73 | 3.56: 1 | 0.22 | *Py1py1py2py2*/*py1py1Py2py2* |
| 18 | 61 | 15 | 76 | 4.07: 1 | 0.86 | *Py1py1py2py2*/*py1py1Py2py2* |
| 19 | 52 | 13 | 65 | 4.00: 1 | 0.33 | *Py1py1py2py2*/*py1py1Py2py2* |
| 20 | 46 | 14 | 60 | 3.29: 1 | 0.02 | *Py1py1py2py2*/*py1py1Py2py2* |
| 21 | 49 | 0 |  |  |  | *Py1Py1py2py2*/*py1py1Py2Py2* |
| 22 | 65 | 0 |  |  |  | *Py1Py1py2py2*/*py1py1Py2Py2* |
| 23 | 57 | 0 |  |  |  | *Py1Py1py2py2*/*py1py1Py2Py2* |
| 24 | 53 | 0 |  |  |  | *Py1Py1py2py2*/*py1py1Py2Py2* |
| 25 | 62 | 0 |  |  |  | *Py1Py1py2py2*/*py1py1Py2Py2* |
| 26 | 46 | 0 |  |  |  | *Py1Py1py2py2*/*py1py1Py2Py2* |
| 27 | 72 | 0 |  |  |  | *Py1Py1py2py2*/*py1py1Py2Py2* |
| 28 | 44 | 0 |  |  |  | *Py1Py1py2py2*/*py1py1Py2Py2* |
| 29 | 58 | 0 |  |  |  | *Py1Py1py2py2*/*py1py1Py2Py2* |
| 30 | 63 | 0 |  |  |  | *Py1Py1py2py2*/*py1py1Py2Py2* |
| 31 | 45 | 0 |  |  |  | *Py1Py1py2py2*/*py1py1Py2Py2* |
| 32 | 57 | 0 |  |  |  | *Py1Py1py2py2*/*py1py1Py2Py2* |

**Table S3** RPKM interval distribution of the 55,250 genes identified in the G-pool and Y-pool via BSR-Seq

| Sample | 0-0.1 | 0.1-1 | 1-3 | 3-15 | 15-60 | >60 |
| --- | --- | --- | --- | --- | --- | --- |
| G-pool | 1,425  (2.58%) | 6,841  (12.38%) | 5,282  (9.56%) | 9,095  (16.46%) | 4,428  (8.01%) | 1,944  (3.52%) |
| Y-pool | 1,456  (2.64%) | 6,740  (12.20%) | 5,344  (9.67%) | 9,172  (16.60%) | 4,468  (8.09%) | 1,936  (3.50%) |

**Table S4** Gene identification and expression data for the DEGs in the Y-pool vs. the G-pool

| Gene ID | log_2_FoldChange | Up-Down-  Regulation | Gene description |
| --- | --- | --- | --- |
| *BraA01000296* | 2.260576363 | Up | Senescence-associated protein |
| *BraA01000441* | 3.459431619 | Up | UDP-glycosyltransferase |
| *BraA01000547* | 1.533929381 | Up | unkown protein |
| *BraA01000705* | 2.455194626 | Up | Flowering-promoting factor 1-like protein |
| *BraA01000852* | 1.44287411 | Up | Putative nuclease Harbi1 |
| *BraA01001526* | 1.59445395 | Up | Mitochondrial uncoupling protein |
| *BraA01003219* | Inf | Up | Non-haem dioxygenase N-terminal domain |
| *BraA01003253* | 3.485426827 | Up | Aspartic proteinase nepenthesin-2 |
| *BraA01003642* | 2.211054877 | Up | Peroxidase |
| *BraA01003858* | 3.618909833 | Up | unkown protein |
| *BraA01004212* | 2.077076223 | Up | Probable mediator of RNA polymerase II transcription subunit |
| *BraA01004585* | 4.266786541 | Up | NAC transcription factor |
| *BraA02000155* | 3.727920455 | Up | 3-ketoacyl-CoA synthase |
| *BraA02001308* | 1.827163403 | Up | Fatty acyl-CoA reductase |
| *BraA02001615* | 4.235216462 | Up | Lipoxygenase 2, chloroplastic |
| *BraA02001616* | 5.700439718 | Up | Lipoxygenase 2, chloroplastic |
| *BraA02001856* | 1.777922268 | Up | Calcium-binding protein PBP1 |
| *BraA02002303* | 1.977482563 | Up | Lipoxygenase 4, chloroplastic |
| *BraA02003902* | 1.500265336 | Up | Putative lipid-transfer protein DIR1 |
| *BraA02003951* | 1.809758747 | Up | Beta-D-xylosidase |
| *BraA02004368* | 2.085167383 | Up | BTB/POZ and TAZ domain-containing protein |
| *BraA03000297* | 3.5360529 | Up | Protein aspartic protease in guard cell |
| *BraA03001067* | 3.297680549 | Up | Ethylene-responsive transcription factor |
| *BraA03001884* | 1.551704339 | Up | Auxin-repressed 12.5 kDa protein |
| *BraA03002224* | 1.443478323 | Up | Nematode resistance protein-like HSPRO2 |
| *BraA03002693* | 2.014075185 | Up | F-box/kelch-repeat protein |
| *BraA03002724* | 3.554588852 | Up | Tricyclene synthase, chloroplastic |
| *BraA03002827* | 1.966734453 | Up | Protein exordium |
| *BraA03003029* | 2.181065559 | Up | Xyloglucan endotransglucosylase/hydrolase protein |
| *BraA03003321* | 2.380423744 | Up | Heat stress transcription factor |
| *BraA03003761* | 3.727920455 | Up | Universal stress protein A-like protein |
| *BraA03004125* | 1.922832139 | Up | unkown protein |
| *BraA03004126* | 3.841302254 | Up | unkown protein |
| *BraA03004153* | 2.070389328 | Up | Zinc finger protein |
| *BraA03004476* | 2.312436646 | Up | Xyloglucan endotransglucosylase/hydrolase |
| *BraA03004719* | 2.062284278 | Up | Two-component response regulator-like APRR1 |
| *BraA03004783* | 1.651530118 | Up | Transcription factor MYB44 |
| *BraA03004969* | 3.421463768 | Up | unkown protein |
| *BraA03006295* | 1.71137388 | Up | Ethylene-responsive transcription factor ERF109 |
| *BraA04000250* | 2.429987841 | Up | AT-hook motif nuclear-localized protein |
| *BraA04002489* | 1.579208973 | Up | BTB/POZ domain-containing protein |
| *BraA04002567* | 1.667424661 | Up | Nematode resistance protein-like HSPRO2 |
| *BraA04002818* | 2.444784843 | Up | B3 domain-containing protein |
| *BraA04002832* | 1.475579041 | Up | E3 ubiquitin-protein ligase |
| *BraA05001058* | 3.285402219 | Up | unkown protein |
| *BraA05002924* | 1.455421345 | Up | Metallothionein-like protein |
| *BraA05002986* | 1.555540692 | Up | Serine/threonine-protein kinase |
| *BraA05003593* | 2.475733431 | Up | WAT1-related protein |
| *BraA05003773* | 1.342145742 | Up | Delta-1-pyrroline-5-carboxylate synthase |
| *BraA05003796* | 2.091404633 | Up | Nematode resistance protein-like HSPRO2 |
| *BraA05003871* | 2.236606281 | Up | MADS-box protein |
| *BraA05004150* | Inf | Up | Protodermal factor |
| *BraA05004240* | 2.801921036 | Up | EGF domain-specific O-linked N-acetylglucosamine transferase |
| *BraA06000244* | 2.061996436 | Up | Abscisic acid 8'-hydroxylase |
| *BraA06000935* | 3.246966037 | Up | Asparagine synthetase |
| *BraA06001540* | 1.724892762 | Up | Heat shock 70 kDa protein |
| *BraA06001801* | 2.383631695 | Up | 1-aminocyclopropane-1-carboxylate oxidase |
| *BraA06003806* | 1.696383783 | Up | Heat stress transcription factor |
| *BraA06004598* | 2.175442006 | Up | Ethylene-responsive transcription factor |
| *BraA07000352* | 3.222392421 | Up | Homeobox-leucine zipper protein |
| *BraA07000827* | 2.055282436 | Up | Pumilio homolog |
| *BraA07001509* | Inf | Up | Extensin-3 |
| *BraA07002255* | 3.639410285 | Up | Myc-type, basic helix-loop-helix (bHLH) domain |
| *BraA07003526* | 3.36923381 | Up | NAC transcription factor |
| *BraA07004133* | 1.622865293 | Up | Caffeoylshikimate esterase |
| *BraA08000847* | 1.795751414 | Up | Aldehyde dehydrogenase family 7 member A1 |
| *BraA08001947* | 2.069540933 | Up | Stem-specific protein |
| *BraA08002747* | 2.833706249 | Up | Gibberellin 2-beta-dioxygenase |
| *BraA08003014* | 3.847996907 | Up | Tetratricopeptide repeat protein |
| *BraA08003163* | 1.915753871 | Up | unknown protein |
| *BraA08003385* | 1.881873899 | Up | Heat shock 70 kDa protein |
| *BraA09000060* | 2.535331733 | Up | Transcription factor HFR1 |
| *BraA09000250* | Inf | Up | Delta-9 acyl-lipid desaturase |
| *BraA09000553* | 2.551409941 | Up | Cytochrome P450 |
| *BraA09000581* | 3.720477471 | Up | 1-aminocyclopropane-1-carboxylate oxidase |
| *BraA09001027* | 2.478047297 | Up | Ethylene-responsive transcription factor |
| *BraA09001276* | 3.048363022 | Up | Ethylene-responsive transcription factor |
| *BraA09001592* | 1.923917607 | Up | Cytochrome P450 |
| *BraA09002229* | 1.762960803 | Up | Basic leucine zipper |
| *BraA09002958* | 2.203942278 | Up | Delta-1-pyrroline-5-carboxylate synthase |
| *BraA09003763* | Inf | Up | Transmembrane protein |
| *BraA09003771* | 1.885195525 | Up | unkown protein |
| *BraA09004641* | 1.317895236 | Up | Polyubiquitin |
| *BraA09005467* | 1.701349075 | Up | Probable acetyl-CoA acetyltransferase |
| *BraA09005587* | 2.364572432 | Up | Abscisic acid 8'-hydroxylase |
| *BraA09006168* | 1.870548749 | Up | Multi antimicrobial extrusion protein |
| *BraA09006663* | Inf | Up | Proline-rich receptor-like protein kinase |
| *BraA09006688* | 1.880888788 | Up | Histone H1 |
| *BraA10000948* | 2.194378045 | Up | Polygalacturonase inhibitor |
| *BraA10002274* | 2 | Up | Transcription factor TRY |
| *BraSca000807* | 2.115343826 | Up | Defensin-like protein |
| *BraA01000216* | -3.196397213 | Down | NEDD8-conjugating enzyme |
| *BraA01001685* | -1.881011964 | Down | Protein IQ-DOMAIN |
| *BraA01003939* | -1.994411762 | Down | Phylloplanin |
| *BraA01003942* | -1.281627823 | Down | Phylloplanin |
| *BraA02000716* | -2.071317405 | Down | Glutamine synthetase |
| *BraA02001393* | -2.574315257 | Down | Germin-like protein |
| *BraA02001524* | -1.517733514 | Down | Amino acid permease |
| *BraA02001889* | -3.099535674 | Down | Probable receptor-like protein kinase |
| *BraA02002017* | -1.552669098 | Down | Zinc finger protein |
| *BraA02003143* | #NAME? | Down | Sulfoquinovosyl transferase |
| *BraA03000231* | -2.514573173 | Down | Leucoanthocyanidin dioxygenase |
| *BraA03000341* | -1.711654506 | Down | Diacylglycerol kinase |
| *BraA03002406* | -1.839535328 | Down | Basic endochitinase |
| *BraA03002411* | -2.009378332 | Down | Basic endochitinase |
| *BraA03003788* | -2.574908836 | Down | Endochitinase |
| *BraA04000359* | -1.429728286 | Down | Glucan endo-1,3-beta-glucosidase |
| *BraA04000670* | -2.68242831 | Down | Glutaredoxin-C14 |
| *BraA04001553* | -4.722466024 | Down | Protein RADIALIS-like |
| *BraA04001995* | -1.486120597 | Down | Ferredoxin-3, chloroplastic |
| *BraA04002500* | -2.026152288 | Down | Monothiol glutaredoxin-S9 |
| *BraA04002600* | -2.641105579 | Down | CASP-like protein |
| *BraA04003328* | -2.049037493 | Down | Glutaredoxin-C13 |
| *BraA05001895* | -1.832890014 | Down | Probable LRR receptor-like serine/threonine-protein kinase |
| *BraA05003213* | -1.273074738 | Down | Defensin-like protein |
| *BraA06001042* | -1.92087135 | Down | Wall-associated receptor kinase |
| *BraA06001968* | -1.827323338 | Down | Probable LRR receptor-like serine/threonine-protein kinase |
| *BraA06002267* | -2.205239474 | Down | High-affinity nitrate transporter |
| *BraA06003167* | -3 | Down | Probable WRKY transcription factor |
| *BraA06003402* | -1.463147513 | Down | Probable xyloglucan endotransglucosylase/hydrolase protein |
| *BraA06003667* | -2.602758084 | Down | Probable inactive purple acid phosphatase |
| *BraA07000474* | -2.614709844 | Down | Ubiquitin-conjugating enzyme |
| *BraA07000874* | -2.060541542 | Down | Adenosylhomocysteinase |
| *BraA07001004* | #NAME? | Down | unkown protein |
| *BraA07001040* | -5.209453366 | Down | Glutathione S-transferase |
| *BraA07001070* | -4.807354922 | Down | Ribosomal protein L32p |
| *BraA07001103* | -2.788495895 | Down | Cystatin-related |
| *BraA07001166* | -1.452368378 | Down | Glucose-6-phosphate 1-dehydrogenase |
| *BraA07001179* | -2.512450001 | Down | Squamosa promoter-binding-like protein |
| *BraA07001255* | -1.609776049 | Down | GDSL esterase/lipase |
| *BraA07001316* | -5.247927513 | Down | Probable LRR receptor-like serine/threonine-protein kinase |
| *BraA07001437* | -1.807354922 | Down | Alcohol dehydrogenase |
| *BraA07001447* | -3.028797264 | Down | unkown protein |
| *BraA07001485* | -3.278535499 | Down | Protein disulfide isomerase |
| *BraA07001632* | -5.459431619 | Down | unkown protein |
| *BraA07003075* | -2.40851578 | Down | WAT1-related protein |
| *BraA07003235* | --2.703018262 | Down | Glycerophosphodiester phosphodiesterase |
| *BraA08002498* | -2.689659879 | Down | Lysine histidine transporter |
| *BraA08003709* | -5.614709844 | Down | High-affinity nitrate transporter |
| *BraA08003844* | -1.558778569 | Down | Two-component response regulator |
| *BraA09000292* | -2.774304956 | Down | Monothiol glutaredoxin |
| *BraA09000392* | -1.949255201 | Down | Calreticulin |
| *BraA09000552* | -1.663747598 | Down | Glucose-6-phosphate/phosphate translocator |
| *BraA09001179* | -5.14974712 | Down | Protein RADIALIS -like |
| *BraA09001661* | -2.187627003 | Down | Expansin-A5 |
| *BraA09002276* | -1.868495217 | Down | Probable pectinesterase |
| *BraA09003323* | -2.022108412 | Down | Uncharacterized protein |
| *BraA09003575* | -2.381429107 | Down | Ureide permease |
| *BraA09003650* | -4.378511623 | Down | unkown protein |
| *BraA09003663* | -3.22650853 | Down | unkown protein |
| *BraA09003718* | -2.342392197 | Down | Ubiquitin-40S ribosomal protein |
| *BraA09004012* | -2.183446141 | Down | CTP synthase |
| *BraA09004189* | -3.05246742 | Down | Heme oxygenase |
| *BraA09004262* | -1.773562748 | Down | unkown protein |
| *BraA09004309* | -1.853710558 | Down | Probable alpha-mannosidase |
| *BraA09004379* | -1.983511877 | Down | unkown protein |
| *BraA09004639* | -2.657718843 | Down | Polyubiquitin |
| *BraA09004917* | -1.874469118 | Down | Elongation factor |
| *BraA09004989* | -3.857980995 | Down | unkown protein |
| *BraA09005402* | -3.117997209 | Down | Defensin-like protein |
| *BraA09005558* | -3.146841388 | Down | Nucleoside diphosphate kinase |
| *BraA09005577* | -1.857980995 | Down | Ras-related protein |
| *BraA09005581* | -3.064130337 | Down | NAC domain-containing protein |
| *BraA09005583* | -2.693896872 | Down | Probable WRKY transcription factor |
| *BraA09005584* | -3.36923381 | Down | Disease resistance protein |
| *BraA09005707* | -7.554588852 | Down | Transcription factor |
| *BraA09005815* | -4.426264755 | Down | Probable LRR receptor-like serine/threonine-protein kinase |
| *BraA09005911* | -2.512450001 | Down | unkown protein |
| *BraA09005918* | -1.833180909 | Down | Serine decarboxylase |
| *BraA09006089* | -2.222392421 | Down | Probable aldo-keto reductase |
| *BraA09006098* | -2 | Down | unkown protein |
| *BraA09006145* | -3.584962501 | Down | unkown protein |
| *BraA09006247* | #NAME? | Down | Ethylene-responsive transcription factor |
| *BraA09006759* | -2.820340564 | Down | Protein nuclear fusion defective |
| *BraA09006920* | -2.678536616 | Down | Subtilisin-like protease |
| *BraA09006921* | -1.698731376 | Down | Subtilisin-like protease |
| *BraA10000293* | -1.52979391 | Down | Glutamine synthetase |
| *BraA10001444* | -1.5363936 | Down | Flavone 3'-O-methyltransferase |
| *BraA10001445* | -1.583463597 | Down | Flavone 3'-O-methyltransferase |
| *BraA10002800* | -2.459431619 | Down | NAC domain-containing protein |
| *BraSca000383* | -3.237039197 | Down | Glycine rich protein |

**Table S5** Primer sequences for the SSR and Indel markers tightly linked with *py1*

| Marker | Forward sequence (5’-3’) | Reverse sequence (5’-3’) |
| --- | --- | --- |
| SSRzk5 | AAATGTGCAAAGAACGCGATA | AACAGATTAGATGAGTCTGATGAACA |
| SSRzk12 | TCAAACCTCTTAAAACCACACA | ATCCGCAAGGGTGTTTCT |
| SSRzk17 | AATAGGATAGTGCTTTTCTTTCATTT | ATACGTTGGTAAGACAGGTTAACATT |
| SSRzk28 | TTAACATGGTGGATCGTAACAGAT | AAAACAAAGAAACAAAAAAGCAAA |
| SSRzk29 | CAAGACGGATTCCAGACAGATT | GAGTTTCGTGCTTTTAGAGAGAGAG |
| SSRzk36 | ACTCTATCCCAAAATTTCACTCCTT | GGACTTGTACTCTGCTTAAAAACCTCT |
| InDelzk125 | AAATCAGTTCTGCTCTTGAGCTTC | GTTTCCTTTTGTTTAGGTTGTGGT |
| InDelzk72 | TAGAAAGAACAAAGTCAGAGGAGA | AAAGAAAATACGTTGATAAACGAG |

**Table S6** Prediction of candidate genes within the gene-mapped region on chromosome A09

| Gene | Start | End | Gene Annotations (BLASTX to *Arabidopsis thaliana*) | E value |
| --- | --- | --- | --- | --- |
| *BraA09004166* | 25,006,409 | 25,007,453 | DNA/RNA-binding protein Alba-like | 3.00E-77 |
| *BraA09004167* | 25,007,714 | 25,015,841 | WD40/YVTN repeat-like-containing domain | 0.00E+00 |
| *BraA09004168* | 25,015,992 | 25,019,712 | DNA/RNA-binding protein Alba-like | 2.00E-111 |
| *BraA09004169* | 25,019,973 | 25,023,544 | WD40/YVTN repeat-like-containing domain | 1.00E-174 |
| *BraA09004170* | 25,032,414 | 25,033,913 | Fructose-1,6-bisphosphatase class 1 | 1.00E-13 |
| *BraA09004171* | 25,046,179 | 25,047,546 | Hypothetical protein | 3.00E-137 |
| *BraA09004172* | 25,048,941 | 25,050,241 | Hypothetical protein | 3.00E-107 |
| *BraA09004173* | 25,055,301 | 25,056,386 | Hypothetical protein | 4.00E-160 |
| *BraA09004174* | 25,063,939 | 25,065,024 | Hypothetical protein | 4.00E-160 |
| *BraA09004175* | 25,081,527 | 25,082,054 | Dof-type zinc finger domain-containing protein | 5.00E-100 |
| *BraA09004176* | 25,086,309 | 25,087,568 | ATS9 (ARABIDOPSIS NON-ATPASE SUBUNIT 9) | 0.00E+00 |
| *BraA09004177* | 25,089,299 | 25,090,366 | Pollen Ole e 1 allergen and extensin family protein | 3.00E-108 |
| *BraA09004178* | 25,114,363 | 25,114,812 | Peptidase C1A papain family protein | 1.00E-96 |
| *BraA09004179* | 25,117,912 | 25,119,046 | Peptidase C1A papain family protein | 1.00E-173 |
| *BraA09004180* | 25,120,829 | 25,121,999 | Peptidase C1A papain family protein | 2.00E-139 |
| *BraA09004181* | 25,129,925 | 25,130,461 | Nucleoside-triphosphatase | 3.00E-70 |
| *BraA09004182* | 25,131,462 | 25,133,561 | Hypothetical protein | 0.00E+00 |
| *BraA09004185* | 25,147,854 | 25,148,988 | Peptidase C1A papain family protein | 2.00E-174 |
| *BraA09004186* | 25,150,769 | 25,151,939 | Peptidase C1A papain family protein | 2.00E-139 |
| *BraA09004187* | 25,171,914 | 25,172,249 | Peptidase C1A papain family protein | 2.00E-39 |
| *BraA09004189* | 25,190,048 | 25,191,179 | Heme oxygenase 1 | 2.00E-134 |
| *BraA09004190* | 25,197,949 | 25,199,064 | Peptidase C1A papain family protein | 2.00E-155 |
| *BraA09004191* | 25,201,347 | 25,202,138 | Ribosomal protein L34 family protein | 1.00E-68 |
| *BraA09004192* | 25,202,395 | 25,203,007 | Hypothetical protein | 2.00E-27 |
| *BraA09004193* | 25,224,380 | 25,227,092 | Hypothetical protein | 0.00E+00 |
| *BraA09004194* | 2,523,0592 | 25,231,880 | Apoptosis inhibitory 5 (API5) family protein | 2.00E-73 |
| *BraA09004197* | 25,240,338 | 25,242,342 | Apoptosis inhibitory 5 (API5) family protein | 6.00E-55 |
| *BraA09004198* | 25,244,443 | 25,246,195 | Apoptosis inhibitory 5 (API5) family protein | 4.00E-52 |
| *BraA09004199* | 25,251,310 | 25,253,838 | Calcium ion binding protein | 0.00E+00 |
| *BraA09004200* | 25,254,452 | 25,256,395 | Hypothetical protein | 4.00E-119 |
| *BraA09004202* | 25,282,583 | 25,283,023 | Hypothetical protein | 1.00E-40 |
| *BraA09004203* | 25,292,796 | 25,293,374 | Kelch repeat-containing F-box family protein | 3.00E-59 |
| *BraA09004204* | 25,294,047 | 25,295,552 | Transferase family protein | 0.00E+00 |
| *BraA09004206* | 25,336,419 | 25,338,049 | Hypothetical protein | 3.00E-40 |

**Table S7** Sequences of the primers used to clone the full-length and CDS sequences of *BraA09004189*

| Markers | Primer Sequences | | Length of PCR products (bp) | Tm (°C) |
| --- | --- | --- | --- | --- |
|  | Forward (5′–3′) | Reverse (5′–3′) |  |  |
| BraA | ACTTTATTCGATCAACTATGGCTTAT | CAAACCCGAAACCACATCAT | 564 | 58 |
| BraB | TTAACTTAAAATGATGTGGTTTCGG | TGAGTAGGATTTTCAAGAGAGAGTG | 674 | 58 |

**Table S8** Primer sequences of the SSR markers tightly linked with *py2*

| Marker | Forward sequence (5’-3’) | Reverse sequence (5’-3’) |
| --- | --- | --- |
| SSR84 | TCTCTTTTGCCAGTGGACGG | AATGGTAAGGAATTCTTATTTACTTTGAA |
| SSR116 | AGGTTCCTTGCTCCTTTGTTT | CTTAGTATTGTCCGCTTTGGG |
| SSR133 | TGATAAGTTAGTAAAAGAATATGGGAAA | CATTGGACGGAGAACAGTTGTAA |
| SSR103 | AGGTGACCGTTATTGATTACAGG | AGGTGGAAACAAGAAAGAAAAGA |
| SSR8 | ATGTAGTTGTACCTTGCTTGCTTA | GTTATTATTTGGACGATGGATTG |
| SSR10 | CTATCGCCATTAGATTGCCACTG | GGCTGACCTTTCTTACCGCTC |
| SSR11 | CTCTTTCCTTTCCTTTTCTTCA | AAGTTTTCTCTTTCTTCTCGTCA |
| SSR15 | ATAAATTGGTGCAAACACATAAA | TAACCACATGAGTAAAAAGTCCC |

**Table S9** Prediction of candidate genes within the gene-mapped region on chromosome A07

| Gene | Start | End | Gene Annotations (BLASTX to *Arabidopsis thaliana*) | E value |
| --- | --- | --- | --- | --- |
| *BraA07001773* | 14,852,848 | 14,855,541 | Zinc ion binding protein | 0.014 |
| *Bra A07001774* | 14,860,138 | 14,862,499 | Emb1187 (embryo defective 1187) | 3.00E-166 |
| *Bra A07001775* | 14,866,992 | 14,868,079 | F-box protein-related F-box | 2.00E-31 |
| *Bra A07001776* | 14,876,973 | 14,878,039 | Zinc ion binding protein | 5.00E-84 |
| *Bra A07001777* | 14,893,414 | 148,936,02 | Unknown protein | 0.00E+00 |

**Table S10** Sequences of the primers used to cloning the full-length sequences of the candidate genes for *py2*

| Markers | Primer sequences | |
| --- | --- | --- |
|  | Forward (5′-3′) | Reverse (5′-3′) |
| BraA07001736 | GGAGTTCCAGGAGACGAGG | GAACACACTCTCTCTTGTCTCCA |
| BraA07001736-2 | GAAGAACGCTATCTCGTGGAC | TCACCAGTAGTCCCCACAAGT |
| BraA07001749 | GCAGCAGAAATAGCAAAGACG | GTCGCTAATCGTCTGGTTGG |
| BraA07001764 | ACGATGACGACCACCAGAA | CTTGGTTCAGGGACGCC |
| BraA07001765-1 | CCAGCAGTAGCAGACAGACC | GAGCAACAAGAAAGGAATCG |
| BraA07001765-2 | AGATGTCTGGAGCATTGGTGT | TCTTCCCGTCTCTGTCTATGTCT |
| BraA07001773-1 | TGGAGGAAGCACTTTCATTG | AGGAAGTGACGGGAAGCAT |
| BraA07001773-2 | GTTTTCTCTTCTCCCTTGGC | AAGAAATGGTGAAGGAAGAAGA |
| BraA07001773-3 | TTCTTCTTCCTTCACCATTTCT | GCTTCTTCTTTCTCCACCATT |
| BraA07001774-1 | AAGTAAGCGGAACCGACCA | AACCTTCAGCACTGCCGTC |
| BraA07001774-2 | GAAAGAAGACAAAGAATCCTCAA | ATCATTGCTACCCATCTCTTG |
| BraA07001775-1 | AACATAACTTCCTCGTCCTGGT | ATCCCGTCCCGCATTTAG |
| BraA07001775-2 | CTCTCGCTTTACTTTCTTTCATC | TTCTAAGTGGAGTAACGCAACA |
| BraA07001775-3 | ACATGATACTTACTTAATGATGGCTT | TGGGAGCAACAATGATGAAG |
| BraA07001776 | TGTGTCTTCTCAGGTTCGGC | CCAATCGCCAGTTTTCCAG |
| BraA07001777 | GGCAGTTGAAGCCGAAGA | GATGAAGTTCCTTTCCCGC |
